# EPIREGULIN creates a developmental niche for spatially organized human intestinal enteroids

**DOI:** 10.1172/jci.insight.165566

**Published:** 2023-03-22

**Authors:** Charlie J. Childs, Emily M. Holloway, Caden W. Sweet, Yu-Hwai Tsai, Angeline Wu, Abigail Vallie, Madeline K. Eiken, Meghan M. Capeling, Rachel K. Zwick, Brisa Palikuqi, Coralie Trentesaux, Joshua H. Wu, Oscar Pellón-Cardenas, Charles J. Zhang, Ian Glass, Claudia Loebel, Qianhui Yu, J. Gray Camp, Jonathan Z. Sexton, Ophir D. Klein, Michael P. Verzi, Jason R. Spence

**Affiliations:** 1 Department of Cell and Developmental Biology, University of Michigan Medical School, Ann Arbor, Michigan, USA.; 2 Department of Computational Medicine and Bioinformatics, University of Michigan, Ann Arbor, Michigan, USA.; 3 Division of Gastroenterology and Hepatology, Department of Internal Medicine, and; 4 Graduate Program in Cellular and Molecular Biology, University of Michigan Medical School, Ann Arbor, Michigan, USA.; 5 Department of Biomedical Engineering, University of Michigan Medical School and University of Michigan College of Engineering, Ann Arbor, Michigan, USA.; 6 Program in Craniofacial Biology and Department of Orofacial Sciences, University of California, San Francisco, San Francisco, California, USA.; 7 New Jersey Medical School, Rutgers, The State University of New Jersey, Newark, New Jersey, USA.; 8 Department of Medicinal Chemistry, College of Pharmacy, University of Michigan, Ann Arbor, Michigan, USA.; 9 Genetic Medicine, Department of Pediatrics, University of Washington, Seattle, Washington, USA.; 10 Department of Materials Science and Engineering, University of Michigan College of Engineering, Ann Arbor, Michigan, USA.; 11 Roche Institute for Translational Bioengineering (ITB), Roche Pharma Research and Early Development, Roche Innovation Center, Basel, Switzerland.; 12 Institute of Molecular and Clinical Ophthalmology Basel, Basel, Switzerland.

**Keywords:** Development, Stem cells, Human stem cells, Organogenesis

## Abstract

Epithelial organoids derived from intestinal tissue, called enteroids, recapitulate many aspects of the organ in vitro and can be used for biological discovery, personalized medicine, and drug development. Here, we interrogated the cell signaling environment within the developing human intestine to identify niche cues that may be important for epithelial development and homeostasis. We identified an EGF family member, EPIREGULIN (EREG), which is robustly expressed in the developing human crypt. Enteroids generated from the developing human intestine grown in standard culture conditions, which contain EGF, are dominated by stem and progenitor cells and feature little differentiation and no spatial organization. Our results demonstrate that EREG can replace EGF in vitro, and EREG leads to spatially resolved enteroids that feature budded and proliferative crypt domains and a differentiated villus-like central lumen. Multiomic (transcriptome plus epigenome) profiling of native crypts, EGF-grown enteroids, and EREG-grown enteroids showed that EGF enteroids have an altered chromatin landscape that is dependent on EGF concentration, downregulate the master intestinal transcription factor *CDX2*, and ectopically express stomach genes, a phenomenon that is reversible. This is in contrast to EREG-grown enteroids, which remain intestine like in culture. Thus, EREG creates a homeostatic intestinal niche in vitro, enabling interrogation of stem cell function, cellular differentiation, and disease modeling.

## Graphical abstract

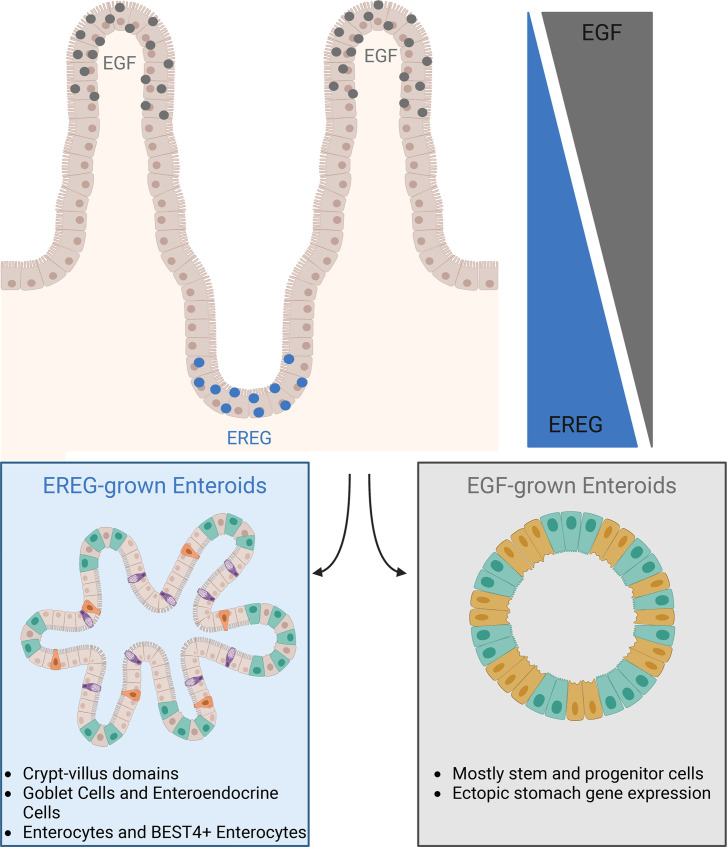

## Introduction

The intestinal epithelium is organized into crypts, where intestinal stem cells (ISCs) are located, and villi, which contain differentiated cell types that carry out absorptive, secretory, and immunologic functions required for life. ISCs are maintained in an environment known as the stem cell niche, which is a collective of biochemical and physical cues that regulate maintenance, self-renewal, and differentiation (1). Niche cues include extracellular matrix, cell-cell contacts, growth factors, cytokines, metabolites, and environmental cues such as food and bacterial products (2). Understanding stem cell regulation and the stem cell niche has been a focus in the murine intestine (3–6), and recent work has begun to interrogate the adult and developing human stem cell niche using single-cell genomic approaches (7–14).

Three-dimensional epithelial organoids, often referred to as enteroids (15–18), have become a powerful in vitro tool to interrogate human stem cell biology and the stem cell niche. First described in mice, enteroids feature crypt-like domains that possess stem and Paneth cells budding from a central lumen that contains differentiated cell types (i.e., goblet, enteroendocrine, enterocyte) in their proper spatial pattern (17, 19–27). Interestingly, while many studies have described the growth/culture of adult and fetal human enteroids, a spatially organized homeostatic state has been difficult to achieve, and instead, human enteroids typically grow as thin-walled cysts replete with proliferative stem and progenitor cells but lacking differentiated cell types and crypt-villus organization (28). Because of this, prior and recent work attempted to identify niche cues that enhance spatial organization and differentiation in adult enteroids (13, 29, 30).

Using single-cell RNA sequencing (scRNA-Seq), we interrogated the stem cell niche in the developing human intestine and leveraged these findings to create a more accurate biomimetic in vitro niche for enteroid growth (31). We identified the EGF family member, EPIREGULIN (EREG), as a growth factor robustly expressed in the stem cell domain of the developing human intestine. Typically, enteroids are grown in the presence of EGF, which is required for proliferation in enteroid culture (22). By replacing the EGF ligand with EREG in enteroid growth media, we observed that enteroids derived from the developing human intestine grew in spatially organized crypt-like and villus-like domains, with the full complement of epithelial cell types found in the native intestine, including rare cell populations such as BEST4^+^ enterocytes (32). Comparing enteroids grown in EGF and EREG using single-nucleus multiomic approaches, we found that EREG-grown enteroids more closely maintained chromatin architecture and gene expression relative to native tissue. In EGF-grown enteroids, global changes in chromatin architecture increased across concentrations of EGF; however, even enteroids grown in low concentrations of EGF lacked spatial organization and differentiation and had ectopic expression of stomach-associated genes, which corresponded to reduced expression of the master intestinal transcription factor, *CDX2*. This phenomenon was reversible if EGF-grown enteroids were switched into EREG-containing media. Collectively, our data show that accurately mimicking the in vivo niche leads to in vitro enteroids that more faithfully model the native intestinal epithelium.

## Results

### Identification of EREG as a niche cue.

**Figure 1:**
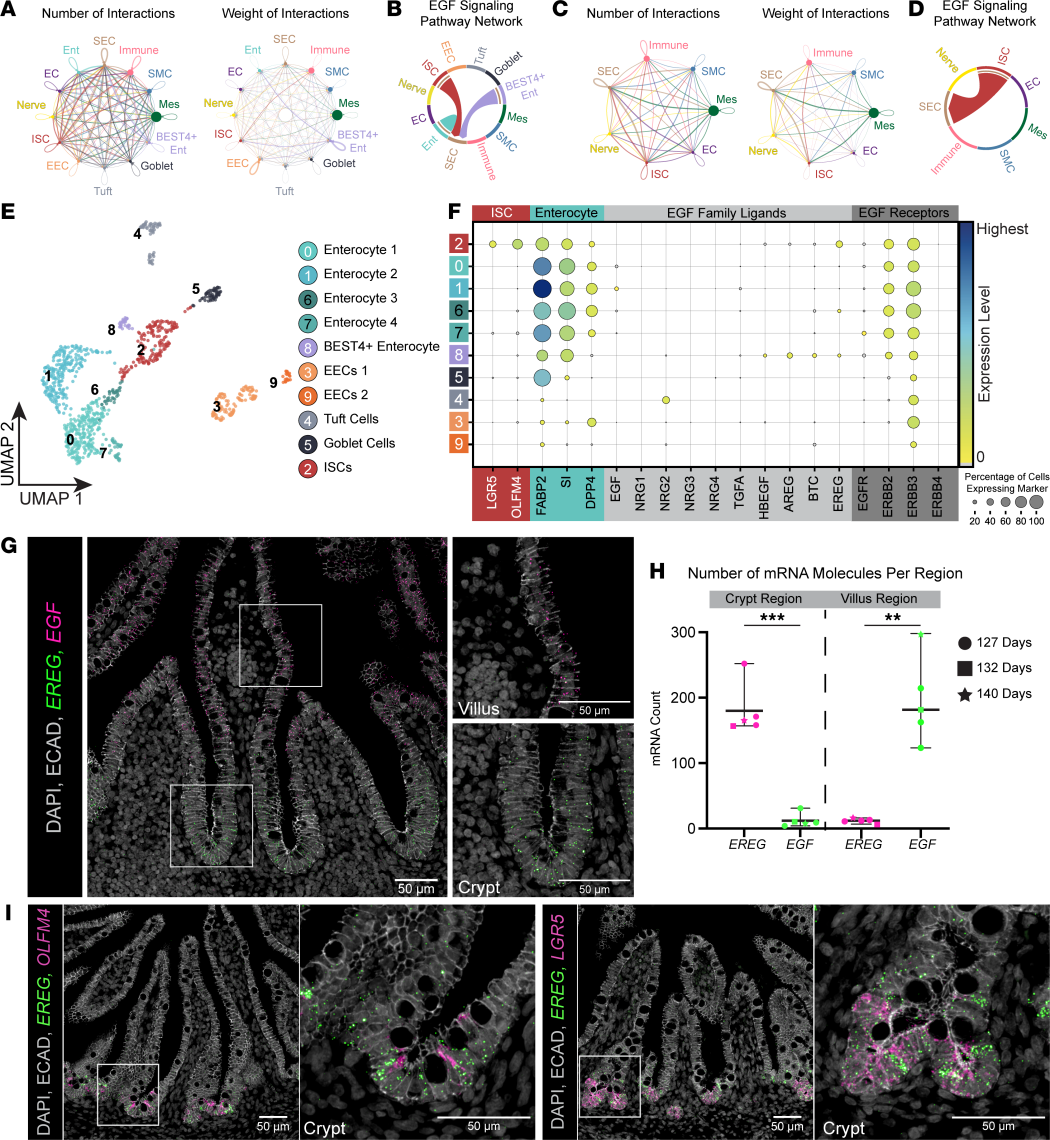
Identification of EREG as a niche cue. (**A**) Aggregated cell-cell communication networks showing the number of interactions (left) or total weighted interaction strengths (right) between all cell types found in 127-day and 132-day fetal tissue single-cell data sets. SEC, subepithelial cells; SMC, smooth muscle cells; Mes, mesenchyme; BEST4^+^ Ent, BEST4^+^ enterocytes; goblet, goblet cells; tuft, tuft cells; EEC, enteroendocrine cells; ISC, intestinal stem cells; EC, endothelial cells; Ent, enterocytes. (**B**) Chord diagram of predicted EGF signaling events throughout the entire data set. (**C**) Aggregated cell-cell communication networks showing the number of interactions (left) or total weighted interaction strengths (right) between a subset of cell types (stem cells and all other cell types) in 127-day and 132-day fetal tissue single-cell data sets. (**D**) Chord diagram of predicted EGF signaling events throughout the subsetted data set. (**E**) UMAP visualization of human fetal small intestinal epithelium (1,009 cells, *n* = 3 biological replicates, 127-day and 132-day). Epithelial cluster identity was determined by expression of canonical lineage markers. (**F**) Dot plot visualization of stem cell markers (*LGR5*, *OLFM4*), enterocyte markers (*FABP2*, *SI*, *DPP4*), EGF ligands, and EGF family receptors among human fetal epithelial data sets. (**G**) Co-FISH/immunofluorescence staining for *EREG* (green), *EGF* (pink), and ECAD (gray) in human fetal duodenum (127-day). Right of main image: zoomed-in images of villus and crypt regions. (**H**) Quantification of EREG and EGF mRNA molecules in crypt and villus regions (*n* = 3 – 127-day specimen, *n* = 1 – 132-day specimen, and *n* = 1 – 140-day specimen). Statistical significance was determined using an unpaired Welch’s 2-tailed *t* test using GraphPad Prism software (crypt significance ***P = 0.0005, villus significance **P = 0.0033). (**I**) Co-FISH/immunofluorescence staining for *EREG* (green), stem cell markers (*LGR5*, *OLFM4*; pink), and ECAD (gray) in human fetal duodenum (127-day).

To better understand the growth factors found in the ISC niche in the fetal human intestine, we leveraged scRNA-Seq data (13, 33) and performed Louvain clustering and visualization using uniform manifold approximation and projection (UMAP) (34). Major cell classes were annotated using cohorts of genes associated with neuronal (*S100B*, *PLP1*, *STMN2*, *ELAVL4*), endothelial (*CDH5*, *KDR*, *ECSCR*, *CLDN5*), mesenchymal (*COL1A1*, *COL1A2*, *DCN*, *ACTG2*, *MYLK*), epithelial (*EPCAM*, *CDH1*, *CDX2*, *CLDN4*), immune (*PTPRC*, *HLA-DRA*, *ARHGDIB*, *CORO1A*), smooth muscle (*ACTA2*, *TAGLN*), and subepithelial cells (*F3*, *PDGFRA*, *NRG1*) (Supplemental Figure 1, D and E; supplemental material available online with this article; https://doi.org/10.1172/jci.insight.165566DS1). Within the epithelial compartment, we identified ISCs (*LGR5/OLFM4*), enterocytes (*FABP2/ALPI/RBP2*), BEST4^+^ enterocytes (*BEST4/SPIB*), goblet cells (*MUC2/SPDEF/DLL1*), tuft cells (*TRPM5/TAS1R3*), and enteroendocrine cells (*CHGA/NEUROD1/PAX6/ARX/REG4*) (Figure 1E and Supplemental Figure 1F).

To interrogate niche signaling within the stem cell compartment, we analyzed these data for receptor-ligand pairing using CellChat (35). We initially interrogated interactions between all the cell types found in the human fetal data set (Figure 1A) and queried specific signaling pathways known to be important for intestinal development and stem cell regulation (Supplemental Figure 1A). This analysis suggested that EGF signaled between the epithelium and underlying subepithelial cell (SEC) compartment (Figure 1B). To examine ISC-niche interactions with more granularity, we further interrogated only stem cell interactions and observed that CellChat predicted only 1 ligand to be signaling from ISCs, *EPIREGULIN* (EREG) (Figure 1, C and D, and Supplemental Figure 1B). CellChat analysis predicted unidirectional EGF signaling from the epithelium to the SEC; however, this prediction is not consistent with functional evidence showing that removal of EGF ligands from human or mouse enteroid culture abrogates proliferation (13, 22). Therefore, we further investigated expression of all members of the EGF family of ligands and receptors in the developing stem cell niche. Consistent with prior reports, we observed expression of the receptors *EGFR* and *ERBB2* throughout the tissue (Supplemental Figure 1C) and *EGF* in enterocytes (Figure 1, E and F) (13), whereas we observed robust expression of *EREG* in ISCs (Figure 1F, cluster 2). Using fluorescence in situ hybridization (FISH), we verified the spatial location of *EREG* in the crypt epithelium of the developing human intestine (Figure 1G). This expression pattern was quantified across 3 different biological samples (Figure 1H). A significant upregulation of *EREG* mRNA was seen in the crypts, while a significant upregulation of *EGF* mRNA was seen on villus cells (Figure 1H). Costaining with stem cell markers *LGR5* and *OLFM4* indicated that both the stem cells and surrounding epithelial cells of the crypt express *EREG* (Figure 1I). We validated these results using 7 independent biological samples from published scRNA-Seq data (13) spanning approximately 7–21 weeks postconception (Supplemental Figure 1, G and H). These additional samples verified the results that *EREG* was enriched in stem cells (Supplemental Figure 1, I and J, cluster 1). FISH staining on several developmental time points further validated these results (Supplemental Figure 1K). This observation suggests that EREG may have a unique role during human intestinal development.

### EREG leads to spatially organized human fetal enteroid cultures.

**Figure 2:**
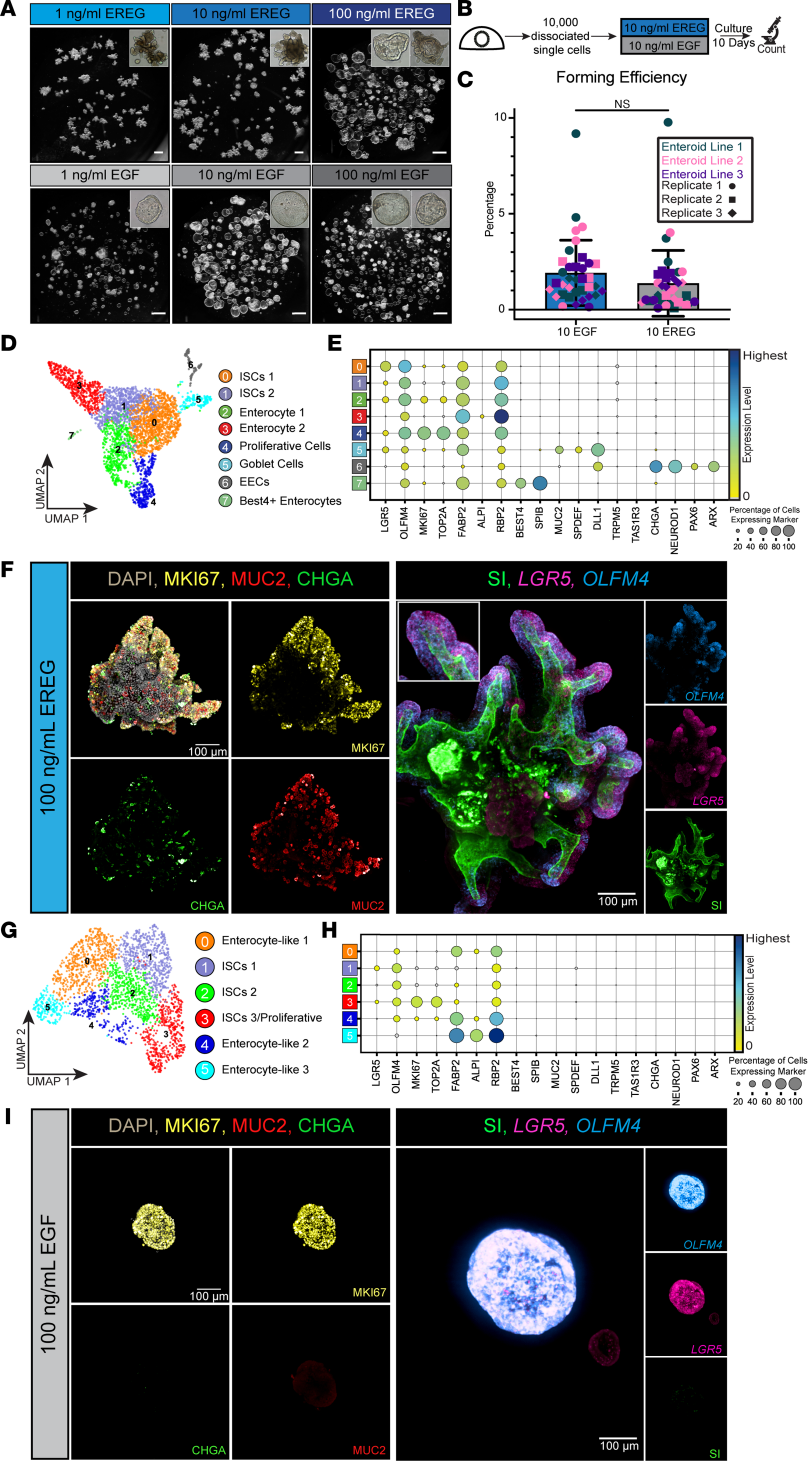
Establishment of spatially organized human epithelial enteroid cultures. (**A**) Bright-field images of enteroids established in various concentrations of EGF or EREG. Scale bar: 500 μm. (**B**) Schematic of experimental design for enteroid forming efficiency assay. (**C**) Enteroid forming efficiency assay results (*n* = 3 biological replicates with *n* = 3 technical replicates quantified at passage 2, passage 3, and passage 4). Statistical significance was determined using an unpaired Welch’s 2-tailed *t* test using the GraphPad Prism software (*P* = 0.1813). (**D**) UMAP visualization of human fetal enteroids established in EREG (1 ng/mL) at passage 1. (**E**) Dot plot visualization for expression of canonical markers of stem cells (*LGR5*, *OLFM4*), proliferative cells (*MKI67*, *TOP2A*), enterocytes (*FABP2*, *ALPI*, *RBP2*), BEST4^+^ enterocytes (*BEST4*, *SPIB*), goblet cells (*MUC2*, *SPDEF*, *DLL1*), tuft cells (*TRPM5*, *TAS1R3*), and enteroendocrine cells (*CHGA*, *NEUROD1*, *PAX6*, *ARX*) in EREG-grown (1 ng/mL) enteroids. (**F**) Whole-mount immunofluorescence (IF) staining (left) of EREG-grown (1 ng/mL) enteroids for the presence of proliferation (MKI67; yellow), and differentiation into goblet cells (MUC2; red), and enteroendocrine cells (CHGA; green). Whole-mount co-FISH/IF (right) for stem cell markers (*LGR5*; pink, *OLFM4*; blue) and brush border of enterocytes (sucrase-isomaltase [SI]; green). (**G**) UMAP visualization of human fetal enteroids established in EGF (100 ng/ mL) enteroids at passage 1. (**H**) Dot plot visualization for expression of canonical markers of stem cells (*LGR5*, *OLFM4*), proliferative cells (*MKI67*, *TOP2A*), enterocytes (*FABP2*, *ALPI*, *RBP2*), BEST4^+^ enterocytes (*BEST4*, *SPIB*), goblet cells (*MUC2*, *SPDEF*, *DLL1*), tuft cells (*TRPM5*, *TAS1R3*), and enteroendocrine cells (*CHGA*, *NEUROD1*, *PAX6*, *ARX*) in EGF-grown (100 ng/mL) enteroids. (**I**) Whole-mount IF staining (left) of EGF-grown (100 ng/mL) enteroids for the presence of proliferation (MKI67; yellow) and differentiation into goblet cells (MUC2; red) and enteroendocrine cells (CHGA; green). Whole-mount co-FISH/IF (right) for stem cell markers (*LGR5*; pink, *OLFM4*; blue) and brush border of enterocytes (SI; green).

To interrogate the role of EREG in the ISC niche, we leveraged human intestinal epithelial-only enteroid cultures derived from the fetal (developing) human intestine for the entirety of this study. We derived these cultures by isolating epithelium from specimens spanning 80–130 days postconception and grew the epithelium in growth media (see Methods) supplemented with varying concentrations of EREG (100 ng/mL, 10 ng/mL, and 1 ng/mL) or matched concentrations of EGF. Media comparison experiments were conducted by isolating tissue from 1 biological specimen and splitting the sample across the required number of media conditions. Therefore, all comparisons within an individual experiment were derived from matched specimens. After 10 days, we documented the gross morphology of these structures. At all concentrations tested, EGF-grown enteroids formed highly proliferative cystic structures as previously reported (36). Conversely, EREG-grown enteroids formed budded structures at lower concentrations, and as protein concentration increased, structures were a mix of budded and cystic enteroids (Figure 2A). We quantified the morphology of enteroids by measuring solidity, aspect ratio, circularity, and roundness for all 6 conditions (Supplemental Figure 2A). We observed that EREG-grown enteroids had lower solidity, circularity, and roundness compared with the cystic EGF-grown enteroids, quantitatively confirming the differences between budded EREG-grown and cystic EGF-grown enteroids. We measured enteroid forming efficiency by performing a quantitative single-cell assay (Figure 2B) and found that EREG-grown enteroids had a lower forming efficiency (1.37%) than EGF-grown enteroids (1.91%), but this was not statistically significant across several biological (*n* = 3) and technical replicates (Figure 2C).

In order to interrogate the cell composition of EREG- and EGF-grown enteroids, we performed scRNA-Seq on both conditions and used well-established genes to identify epithelial composition of both samples. We also carried out 2D and 3D imaging to characterize cellular localization and spatial organization of enteroids derived from both growth conditions. scRNA-Seq data from EREG-grown enteroids demonstrated that all cell types found in the epithelium of the developing human intestine (Figure 1) were present, including ISCs (*LGR5/OLFM4/MKI67*), goblet cells (*MUC2/SPDEF/DLL1*), enteroendocrine cells (*CHGA/NEUROD1/PAX6/ARX/REG4*), and BEST4^+^ enterocytes (*BEST4/SPIB*) (Figure 2, D and E). However, EGF-grown enteroids lacked expression of most major cell types and were instead composed mostly of stem cells and progenitor cells beginning to differentiate into enterocyte-like cells (Figure 2, G and H). Whole-mount IF staining of EREG-grown enteroids demonstrated the presence of goblet and enteroendocrine cells throughout the budded structures (Figure 2F) consistent with in vivo localization (Supplemental Figure 3, A and E). Enterocytes, identified by the brush border enzyme SI, lined the apical surface of the central lumen. Stem cell genes (*OLFM4*, *LGR5*) and the proliferation marker (MKI67) were restricted to the budded regions, suggesting that these structures are crypt-like domains (Figure 2F). Observations from 3D imaging were confirmed in thin sections via FISH/IF and via transmission election microscopy (Supplemental Figure 3, B and F). EGF-grown enteroids, conversely, lacked expression of goblet, enteroendocrine, and brush border markers, but had MKI67 staining throughout enteroids, corresponding to ubiquitous expression of stem cell markers (*OLFM4*, *LGR5*) (Figure 2I and Supplemental Figure 2C). Neither culture condition produced Paneth cells, which normally begin to appear in the developing human intestine after 21–22 weeks postconception (37) and can be cultured in adult systems (30).

While all doses of EGF-grown enteroids grew in a cystic morphology, we wanted to ensure that lower dosages of EGF formed comparable enteroids on a more granular level. We leveraged multiomic sequencing on 1 ng/mL EGF-grown enteroids and 100 ng/mL EGF-grown enteroids and found that when projecting their transcriptome and epigenome in a weighted nearest neighbors (WKNN) graph, these 2 concentrations completely overlapped (Supplemental Figure 2B). Looking closer at their transcriptome, 100 ng/mL EGF and 1 ng/mL EGF samples overlapped on a UMAP graph and similarly featured cell populations composed mainly of stem cells and some enterocyte-like cells (Supplemental Figure 2, C–E). Similarly, enteroids grown in 1 ng/mL EGF had few differentiated cells (Supplemental Figure 2, F and G), like enteroids grown in 100 ng/mL EGF (Figure 2, G and H). These findings were further validated with FISH/IF staining (Supplemental Figure 3D) and suggest at a protein level, 1 ng/mL EGF are indistinguishable from 100 ng/mL EGF enteroids. Enteroids grown in either concentration of EGF lacked differentiated cell types. Therefore, since these 2 doses were virtually identical in morphology, transcriptome, and epigenome, we focused our analysis on the higher concentration of 100 ng/mL EGF since this concentration is most commonly used in the field. These data collectively show that standard EGF conditions lead to poorly differentiated enteroids enriched in stem cells, while EREG-grown enteroids recapitulate the spatial organization and cellular diversity of the developing human intestine in vitro.

### EGF enteroid cultures ectopically express gastric epithelial genes.

**Figure 3:**
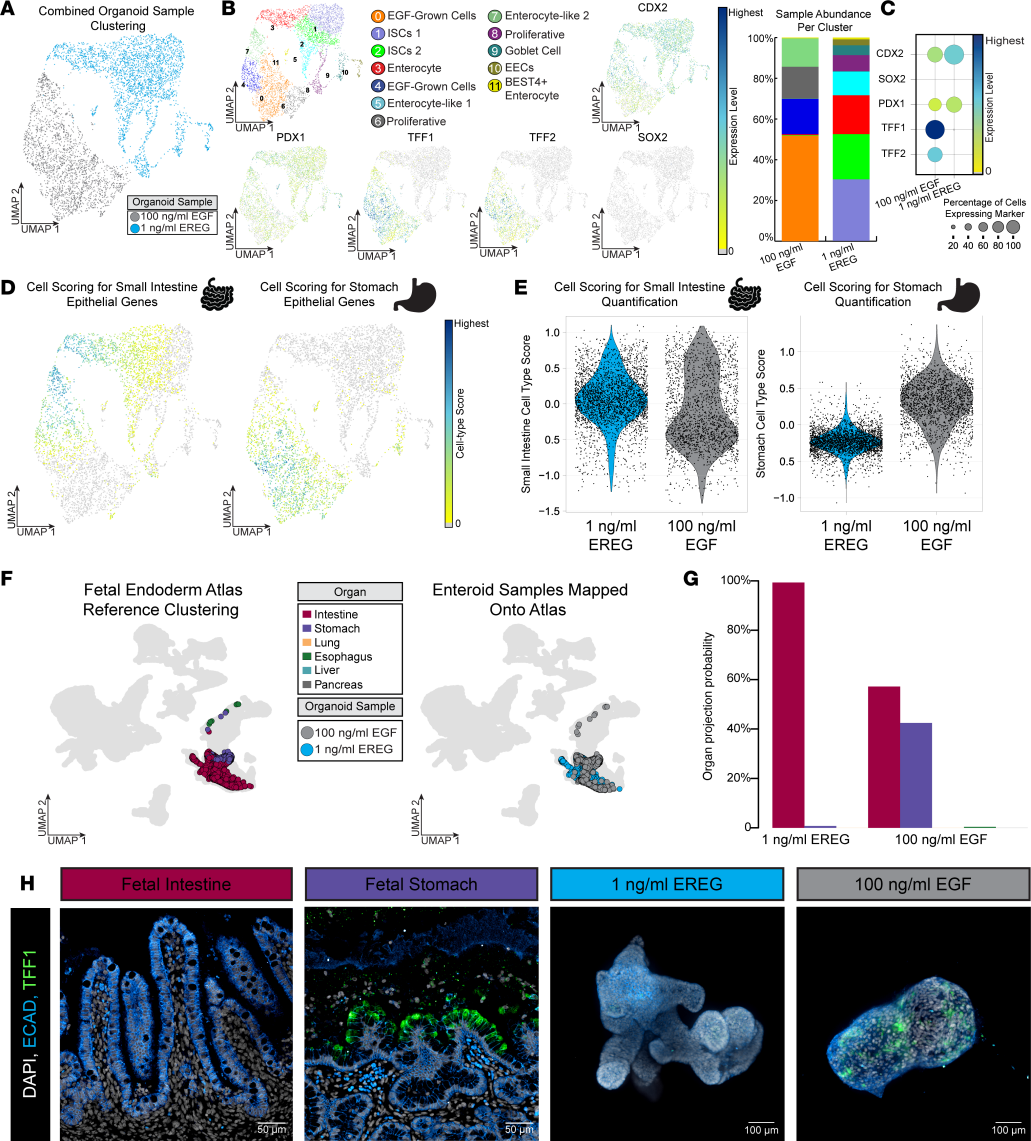
EGF-grown enteroids ectopically express gastric epithelial genes. (**A**) UMAP visualization of EREG-grown (1 ng/mL) enteroids (blue) and EGF-grown (100 ng/mL) enteroids (gray). (**B**) UMAP visualization of overall Louvain clustering and gene expression of *CDX2*, *SOX2*, *PDX1*, *TFF1*, and *TFF2*. Bar plot quantification of cell type abundance per Louvain cluster in each sample; colors correspond to color of cluster. (**C**) Dot plot quantification of gene expression shown in **B**, grouped by culture condition. (**D**) UMAP visualization of cell scoring analysis for small intestine genes (left) and stomach genes (right); see Supplemental Table 2 for gene list. (**E**) Violin plot quantification of cell type score as shown in **D**. (**F**) UMAP visualization of reference fetal atlas organ tissues (12) (left) and enteroid samples embedding onto reference map (right); blue dots represent 1 ng/mL EREG-grown cells, and gray dots represent 100 ng/mL EGF-grown cells. (**G**) Quantification of **F**. Bars represent the percentage of each sample that map to a specific tissue type. (**H**) Immunofluorescence staining for stomach gene TFF1 in human fetal intestine (127-day) and stomach (132-day), 1 ng/mL EREG-grown enteroids (passage 1 day 10), and 100 ng/mL EGF-grown enteroids (passage 1 day 10).

After analyzing scRNA-Seq data sets for EREG- or EGF-grown enteroids independently (Figure 2), we next integrated these data in order to more directly compare differences in these culture conditions. We observed that the 2 samples clustered separately (Figure 3A and Supplemental Figure 4, A and B), and the majority of cells occupied distinct clusters within the UMAP embedding (Figure 3B). We investigated differentially expressed genes (Supplemental Table 1) and noticed higher expression of genes that are typically associated with the gastric epithelium, including TFF1 and TFF2 (38, 39), in the EGF-grown enteroids (Figure 3, B and C; and expanded gastric gene list, Supplemental Figure 4C). To interrogate this finding further, we compiled a cohort of 50 genes that are highly expressed in the developing stomach epithelium and 50 genes that are highly expressed in the developing intestinal epithelium based on a recently published Human Fetal Endoderm Atlas (33). This allowed us to generate a “score” for each cell in each sample based on the enrichment of stomach or intestinal gene sets. This analysis demonstrated that the EGF-grown enteroids scored more highly for the stomach gene cohort, and the EREG-grown enteroids scored more highly for the small intestinal gene cohort (Figure 3, D and E, and Supplemental Table 2). To validate this finding using a different, but complementary, computational approach, we mapped single cells from EGF- and EREG-grown enteroids onto the entire Human Fetal Endoderm Atlas (esophagus, stomach, small and large intestine, lung, and liver) and found that a large proportion of cells from EGF-grown enteroids mapped to the gastric epithelium, while EREG-grown samples primarily mapped to the small intestinal epithelium (Figure 3F). Mapping was quantified (Figure 3G) and we found that EREG-grown enteroids mapped almost exclusively to the small intestine, but EGF-grown enteroids had approximately 40% of cells that mapped to the gastric epithelium, with a small number of cells mapping to the esophagus. Immunofluorescence staining for individual stomach markers such as TFF1 (Figure 3H and Supplemental Figure 4, D and E) and CLDN18 (Supplemental Figure 4, E and F) verified ectopic stomach expression throughout EGF-grown enteroids.

### EGF enteroid cultures have reduced CDX2 expression and an altered chromatin landscape, corresponding to ectopic stomach gene expression.

**Figure 4:**
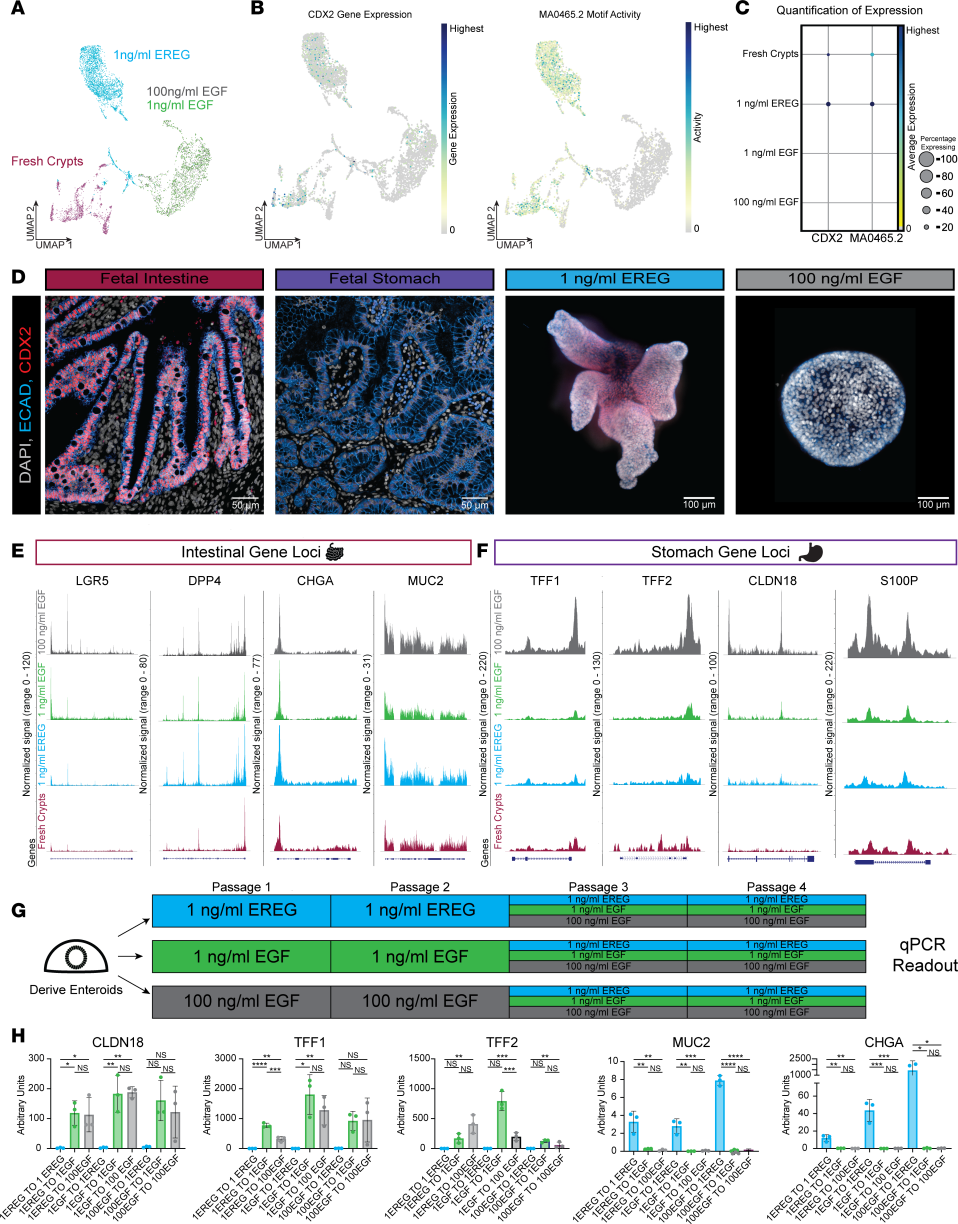
Multiomic analysis of EGF- and EREG-grown enteroids suggests altered chromatin landscape. (**A**) Weighted nearest neighbors (representing joint RNA and ATAC data) UMAP visualization colored by sample (fresh crypts; red, 1 ng/mL EREG [passage 0 day 10]; blue, 1 ng/mL EGF [passage 0 day 10] green; 100 ng/mL EGF [passage 0 day 10]; gray). (**B**) UMAP visualization of *CDX2* gene expression (left) and corresponding motif activity (right). (**C**) Dot plot quantification of *CDX2* gene expression and motif activity (MA0465.2). (**D**) Whole-mount and 2D immunofluorescence staining for CDX2 in fetal intestine (127-day), fetal stomach (132-day), 1 ng/mL EREG-grown enteroid (passage 1 day 10), and 100 ng/mL EGF-grown enteroid (passage 1 day 10). (**E**) Chromatin accessibility of intestinal genes including *LGR5*, *DPP4*, *MUC2*, and *CHGA*. (**F**) Chromatin accessibility of stomach genes including *TFF1*, *TFF2*, *CLDN18*, and *S100P*. (**G**) Schematic of ligand switching experiment. (**H**) RT-qPCR quantification of stomach markers (*CLDN18*, *TFF1*, and *TFF2*) and intestinal secretory cells (*CHGA*, *MUC2*) in enteroids grown in EGF or EREG for 2 passages, then switched to opposite ligand for 2 additional passages. Please see Methods for further details. One-way ordinary ANOVA with multiple comparisons. *: *P* ≤ 0.05; **: *P* ≤ 0.01; ***: *P* ≤ 0.001; ****: *P* ≤ 0.0001.

To further understand how EGF or EREG culture conditions are affecting ISCs, we isolated fresh crypts from human fetal tissue, snap-froze a portion of the sample immediately, and cultured the rest of the sample in either 1 ng/mL EREG or EGF (at 1 ng/mL and 100 ng/mL, respectively) for 1 passage. We then isolated nuclei and subjected all 4 samples to 10x Genomics multiomic sequencing to observe changes in gene expression and the chromatin landscape and to directly compare culture conditions with those of the native crypt. A WKNN graph, which represents a weighted combination of RNA- and ATAC-Seq modalities, was used to cluster cells (40), and visualization of sample distribution was carried out via UMAP (Figure 4A). We also interrogated the multiomic data to identify differentially expressed transcription factors and to identify differentially enriched motifs within the chromatin accessibility data. This analysis revealed that several transcription factors known to be critical for intestinal development were differentially expressed, including *CDX2*, *GATA4*, and *HNF4A* (41, 42), and genes with motifs for these transcription factors were downregulated in EGF-grown enteroids when compared with fresh crypts and EREG-grown enteroids (Figure 4, B and C, and Supplemental Figure 5, A and B).

IF whole-mount staining (Figure 4) or staining of sections (Supplemental Figure 5) of EGF- and EREG-grown enteroids for CDX2 protein showed that EGF-grown enteroids had a lower level of CDX2 protein expression as compared with EREG-grown enteroids (Figure 4D and Supplemental Figure 5C).

To interrogate global chromatin accessibility changes between groups, we treated single-nucleus ATAC-Seq (snATAC-Seq) data as pseudo-bulk data and applied k-means clustering to interrogate patterns of accessibility between samples (Supplemental Figure 5, D and E). We found that EREG-grown enteroids maintained many regions of open chromatin similar to in vivo crypts, and these regions were also closed in 100 ng/mL EGF-grown enteroids (i.e., clusters 3 and 5) with 1 ng/mL EGF-grown enteroids having an intermediate average peak size. In general, 1 ng/mL EGF-grown organoids possessed accessibility peak intensities that were in between 1 ng/mL EREG and 100 ng/mL EGF, both when chromatin accessibility was gained (Supplemental Figure 5, D and E, clusters 2 and 4) and when chromatin accessibility was lost (Supplemental Figure 5, D and E, cluster 3). Of note, when we performed DNA motif enrichment analysis, we observed that cluster 3 and cluster 5 were enriched for well-established intestinal transcription factor motifs, including CDX2 (43), GATA4, and HNF4A (44) (Supplemental Figure 5F and Supplemental Table 3). We also observed a large group of genomic loci (cluster 4) with a gain-of-accessibility in the EGF-grown enteroids. Analysis of motifs enriched at these EGF-accessible chromatin regions revealed motifs for FRA1, which is a transcription factor associated with gastric cancer (41), as well as for SOX2 (42), which is expressed in the early foregut. Chromatin accessibility for cluster 4 remained low in EREG-grown enteroids, though levels were slightly elevated relative to in vivo crypts (Supplemental Figure 5, D–F). We also examined the chromatin accessibility for individual gene loci associated with different epithelial cell types, including *LGR5* (stem cells), *CHGA* (enteroendocrine cells), *DPP4* (enterocytes), and *MUC2* (goblet cells) and observed few differences between conditions (Figure 4E). Since we observed ectopic stomach gene expression in the EGF enteroids (Figure 3), we interrogated stomach gene loci in the snATAC-Seq data as well. As predicted by expression data, and consistent with global trends in genome accessibility (Supplemental Figure 5, D and E), we found stomach gene loci with enhanced accessibility in EGF-grown enteroids, in a dose-dependent manner (Figure 4F). For example, *TFF1*, *TFF2*, *CLDN18*, and *S100P* all had enhanced accessibility in 100 ng/mL EGF, while only *TFF2* and *CLDN18* had modest increases in accessibility at 1 ng/mL EGF compared with EREG. Collectively, these data suggest that while EGF drives ectopic stomach gene expression in enteroids, it does not induce a loss of intestinal identity or the complete loss of chromatin accessibility at intestinal loci (Figure 4F).

Based on our observations, we reasoned that the ectopic expression of gastric genes induced by EGF may be reversible. To test this possibility, we generated enteroids in 1 ng/mL EREG, 1 ng/mL EGF, or 100 ng/mL EGF and grew them for 2 passages. Upon third passage, we switched the media from EREG-to-EGF or EGF-to-EREG (Figure 4G). Using reverse transcription quantitative PCR (RT-qPCR) as a readout, we found that enteroids switched into EREG irrespective of their starting EGF concentration downregulated stomach-associated genes (*CLDN18*, *TFF1*, *TFF2*) and upregulated goblet cell (*MUC2*) or enteroendocrine cell (*CHGA*) markers, which are normally absent when grown in EGF media (Figure 2, G–I). EREG-grown enteroids showed the opposite gene expression profile, where enteroids switched into EGF upregulated stomach-associated genes (*CLDN18*, *TFF1*, *TFF2*) and downregulated *MUC2* and *CHGA* (Figure 4H). These findings were consistent irrespective of the starting or ending EGF concentration. Taken together, these data show that EGF leads to increased accessibility at stomach gene loci, and expression of bona fide stomach genes with a paucity of differentiated intestinal epithelial cell types, while EREG*-*grown enteroids are spatially organized with fully differentiated cell types, maintain high CDX2 expression, and more faithfully recapitulate the small intestine in vitro.

## Discussion

Here we describe an enteroid culture system derived from human fetal intestinal samples that creates spatially organized enteroids with most of the epithelial cell types found in vivo. We identified EREG as a stem cell niche cue unique to the developing human intestine and demonstrated that fetal enteroids grown in EREG led to gene expression and a chromatin accessibility signature that remained more faithful to the native crypt epithelium compared with EGF. Our efforts to define a more physiologic and biomimetic artificial in vitro niche are in line with ongoing efforts by us and by others (13, 29, 30). For example, a recent study defined conditions that allowed spatially organized growth and differentiation of Paneth cells in adult human enteroids and showed that addition of IL-22 boosted Paneth cell numbers in these cultures (30). In adult murine enteroid cultures, Paneth cell differentiation is a critical event for stem cell symmetry breaking and the formation of organized crypt-villus units (20). It is interesting to note that the fetal intestine and fetal enteroids examined in this study do not yet possess Paneth cells, and so the niche cues for appropriately culturing and maintaining fetal (i.e., EREG) or adult (i.e., IL-22) enteroids will need to be tailored for each unique biological context.

We also observed that the widely used EGF culture system causes ectopic stomach gene expression. We find that the shift in chromatin landscape caused by EGF in vitro is dose dependent and is associated with a decrease in *CDX2* expression. These observations are consistent with prior studies examining this master intestinal transcription factor, which causes a complete loss of intestinal identity when genetically deleted in the early murine intestine (45), or, leads to the ectopic expression of stomach genes when deleted from the intestine at later stages of development or in the adult (43).

It is important to point out that while EGF-grown enteroids had reduced CDX2 expression and expressed stomach genes, this did not correspond to a complete loss of CDX2 or an increase in the foregut/stomach transcription factor SOX2. Additionally, when switching enteroids between EGF and EREG, we found that stomach-associated gene expression was reversible, suggesting that EGF is not inducing a true homeotic transformation into stomach epithelium. Rather, our results suggest that the intestinal identity is maintained in EGF-grown enteroids, but they also show that growth factors from the same family (i.e., EGF, EREG) do not always elicit the same biological effect and that more faithfully recapitulating the in vivo stem cell niche yields a better in vitro intestinal mimic.

## Methods

### Microscopy

All fluorescence images were taken on a Nikon A1 confocal microscope for 2D sections, an Olympus IX83 fluorescence microscope for bright-field images, or a Nikon X1 Yokogawa Spinning Disk/Yokogawa CV8000 microscope for 3D whole-mount images. Transmission electron microscopy (TEM) sample grids were imaged on a JEOL JEM 1400 PLUS TEM. Acquisition parameters were kept consistent within the same experiment, and all image post-processing was performed equally across all images of the same experiment. Images were assembled in Adobe Photoshop CC 2022.

### Tissue processing, staining, and quantification

#### Tissue processing.

After harvest, tissue was immediately fixed in 10% Neutral Buffered Formalin (NBF) for 24 hours at RT on a rocker. Tissue was then washed 3 times in UltraPure DNase/RNase-Free Distilled Water (Thermo Fisher Scientific, catalog 10977015) for 30–60 minutes per wash depending on sample size. Next, tissue was dehydrated through an alcohol series diluted in UltraPure DNase/RNase-Free Distilled Water for 30–60 minutes per solution: 25% MeOH, 50% MeOH, 75% MeOH, 100% MeOH. Tissue was either immediately processed into paraffin blocks or stored long term at 4°C. Immediately before paraffin processing, dehydrated tissue was placed in 100% EtOH, followed by 70% EtOH, and perfused with paraffin using an automated tissue processor (Leica ASP300) with 1 hour solution changes overnight. Prior to sectioning, the microtome and slides were sprayed with RNase Away (Thermo Fisher Scientific, catalog 700511). Sections, 5 μm thick, were cut from paraffin blocks onto charged glass slides. Slides were baked for 1 hour in a 60°C dry oven (within 24 hours of performing FISH or within a week for IF). Slides were stored at room temperature (RT) in a slide box containing a silica desiccator packet and the seams sealed with paraffin.

#### Antibody and FISH probe information.

All antibodies were used on FFPE processed sections as described above; no frozen sections were used. The following antibodies were used throughout the manuscript in IF and FISH with co-immunofluorescence staining: rabbit anti-MKi67 1:200 (Thermo Fisher Scientific catalog RM-9106-S1), goat anti-CHGA 1:100 (Santa Cruz Biotechnology catalog sc-1488), mouse anti-CDX2 1:500 (BioGenex catalog AM392), rabbit anti-Mucin2 (1:50 Santa Cruz Biotechnology catalog sc-15334), goat anti-E-Cadherin 1:500 (R&D Systems catalog AF748), mouse anti-E-Cadherin 1:500 (BD Transduction Laboratories catalog 610181), mouse anti-SI 1:50 (Santa Cruz Biotechnology catalog sc-393424), mouse anti-TFF1 (1:100 Abcam catalog ab268118), and rabbit anti-CLDN18 (1:1,000 Abcam catalog ab203563). The antigen retrieval step for the following antibodies was completed in a steamer: Ki67, CHGA, MUC2, ECAD, and SI. The antigen retrieval step for the following antibodies was completed in a pressure cooker: CDX2, TFF1, and CLDN18.

FISH probes were acquired from ACD and stained using the RNAscope multiplex fluorescent manual protocol and kit (ACD, RNAscope Multiplex Fluorescent Reagent Kit v2): RNAscope Probe Hs-LGR5 (ACD catalog 311021), RNAscope Probe Hs-OLFM4 (ACD catalog 311041), RNAscope Probe Hs-EREG (ACD, catalog 313081), RNAscope Probe Hs-EGF (ACD 605771), RNAscope Probe Hs-EGFR (ACD 310061), and RNAscope Probe Hs-ERBB2-C2 (ACD 310081-C2).

#### IF protein staining on paraffin sections.

Tissue slides were rehydrated in Histo-Clear II (National Diagnostics, catalog HS-202) twice for 5 minutes each, followed by serial rinses through the following solutions twice for 2 minutes each: 100% EtOH, 95% EtOH, 70% EtOH, 30% EtOH, and finally double-distilled water (ddH_2_O) 2 times for 5 minutes each. Antigen retrieval was performed using 1× sodium citrate buffer (100 mM trisodium citrate (MilliporeSigma, catalog S1804) and 0.5% Tween 20 (Thermo Fisher Scientific, catalog BP337) at pH 6.0, steaming the slides for 20 minutes, then washing quickly twice in ddH_2_O. Slides were incubated in a humidity chamber at RT for 1 hour with blocking solution (5% normal donkey serum [MilliporeSigma, catalog D9663] in PBS with 0.1% Tween 20). Slides were then incubated in primary antibody diluted in blocking solution at 4°C overnight in a humidity chamber. Next, slides were washed 3 times in 1× PBS for 5 minutes each and incubated with secondary antibodies with DAPI (1 μg/mL) diluted in blocking solution for 1 hour at RT in a humidity chamber. Slides were then washed 3 times in 1× PBS for 5 minutes each and mounted with ProLong Gold (Thermo Fisher Scientific, catalog P369300) and imaged within a week. Stained slides were stored in the dark at 4°C. Secondary antibodies were purchased from Jackson ImmunoResearch and used at a dilution of 1:500, including AffiniPure Donkey anti-Mouse IgGAF488/Cy3/AF647 (catalog 115-545-003, 115-165-003, 115-605-003), AffiniPure Donkey anti-Goat IgGAF488/Cy3/AF647 (catalog 705-545-003, 705-165-003, 705-605-003), and AffiniPure Donkey anti-Rabbit IgGAF488/Cy3/AF647 (catalog 111-545-003, 111-165-003, 111-605-003).

#### FISH on paraffin sections.

The FISH protocol was performed according to the manufacturer’s instructions (ACD) with a 30-minute protease treatment and 15-minute antigen retrieval. For IF protein costains, the DAPI counterstaining final step of the FISH protocol was skipped, and instead, the slides were washed 3 times for 5 minutes in PBS followed by the IF protocol above from the blocking stage onward.

#### Whole-mount IF with antibody staining.

All tips and tubes were coated with 1% BSA in PBS to prevent tissue loss. Enteroids were dislodged from Matrigel using a blunt tip P1000 and transferred to a 1.5 mL Eppendorf tube. A total of 500 μL of Cell Recovery Solution (Corning, catalog 354253) was added to the tube and placed on a rocker at 4°C for 50 minutes to completely dissolve Matrigel. Tubes were spun at 100*g* for 5 minutes at 4°C, after which the solution and remaining Matrigel were removed. Tissue was fixed in 10% NBF overnight at RT on a rocker. The next day, tissue then was washed 3 times for 2 hours with 1 mL of organoid wash buffer (OWB) (0.1% Triton, 0.2% BSA in 1× PBS) at RT on a rocker. Wash times varied (30 minutes – 2 hours) depending on tissue size. A total of 1 mL of CUBIC-L (TCI Chemicals catalog T3740) was added to the tube and placed on a rocker for 24 hours at 37°C. Tissue was washed 3 times with 1 mL of OWB, then permeabilized for 24 hours at 4°C on a rocker with 1 mL permeabilization solution (5% normal donkey serum, 0.5% Triton in 1× PBS). After 24 hours, permeabilization solution was removed, and 500 μL primary antibody (diluted in OWB) was added overnight at 4°C on a rocker. The next day, tissue was washed 3 times with 1 mL of OWB, 2 hours each at RT. Then 500 μL of secondary antibody (diluted in OWB at a 1:500 dilution) was added and incubated overnight at 4°C, wrapped in foil. Tissue was washed again 3 times with 1 mL OWB at RT, first wash for 2 hours with an added DAPI dilution of 1:1,000, then 30 minutes for the remaining washes. Samples were transferred to a 96-well imaging plate (Thermo Fisher Scientific catalog 12-566-70) and then cleared and mounted with 50 μL CUBIC-R (just enough to cover tissue) (TCI Chemicals catalog T3741). Whole-mount images were taken on a Nikon X1 Yokogawa Spinning Disk Microscope or the Yokogawa CV8000 microscope.

#### Whole-mount FISH with antibody staining.

All tips and tubes were coated with 1% BSA in PBS to prevent tissue loss. Enteroids were dislodged from Matrigel using a blunt tip P1000 and transferred to 1.5 mL Eppendorf tube. A total of 500 μL of Cell Recovery Solution (Corning catalog 354253) was added to the tube and placed on a rocker at 4°C for 50 minutes to completely dissolve Matrigel. The tube was spun at 100*g* for 5 minutes at 4°C, after which the solution and remaining Matrigel were removed. Tissue was fixed in 10% NBF overnight at RT on a rocker. Tissue was washed 3 times for 30 minutes at RT with RNase-free water and then dehydrated using a methanol series of 25% MeOH in PBS with 1% Tween (PBST), 50% MeOH in PBST, and 75% MeOH in PBST, and enteroids were stored in 100% MeOH at –20°C for up to 6 months. Enteroids were then rehydrated using the same methanol gradient in reverse (75% MeOH in PBST, 50% MeOH in PBST, and 25% MeOH in PBST) and washed in PBS for the final wash. Enteroids were moved to an imaging plate for the remainder of the protocol. Using the RNAscope Multiplex Fluorescent Reagent Kit v2, 3 drops of H_2_O_2_ were added at RT for 10 minutes on a shaker. Supernatant was aspirated and sample was washed 3 times with ddH_2_0 for 5 minutes each wash. A total of 100 μL of diluted protease III (1:15 in PBS) was added for 10 minutes at 40°C in a humidity chamber. Protease was removed and sample was washed 3 times in PBS for 5 minutes each. Probe was added and incubate for 2 hours at 40°C in the humidity chamber. Samples were washed twice for 5 minutes in RNAscope wash buffer. From here, the remainder of the protocol was performed according to the manufacturer’s instructions (ACD). To add an IF protein costain, tissue then was washed quickly 3 times with 1× PBST at RT on a rocker, wrapped in aluminum foil. Tissue was blocked with 5% normal donkey serum in 0.1% PBST for 1 hour at RT on a rocker. A total of 500 μL primary antibody (diluted in OWB [0.1% Triton, 0.2% BSA in 1× PBS]) was added overnight at 4°C on a rocker. The next day, tissue was washed 3 times with 1 mL of OWB, 2 hours each at RT. Wash times varied (30 minutes to 2 hours) depending on tissue size. A total of 500 μL of secondary antibody (diluted in OWB at a 1:500 dilution) was added and incubated overnight at 4°C, wrapped in foil. Tissue was washed again 3 times with 1 mL OWB at RT, first wash for 2 hours with an added DAPI dilution of 1:1,000, then 30 minutes for the remaining washes. Samples were transferred to a 96-well, glass-bottom imaging plate (Thermo Fisher Scientific 12-566-70) and then cleared and mounted with 50 μL CUBIC-R (just enough to cover tissue). Whole-mount images were taken on a Nikon X1 Yokogawa Spinning Disk Microscope or the Yokogawa CV8000 microscope.

#### TEM.

EREG-grown enteroids were cultured for 1 passage, and small sections of whole intestine from 89 days postconception human fetal tissue were collected for TEM and prepared using conventional TEM sample preparation methods described by the University of Michigan BRCF Microscopy and Image Analysis Laboratory. Samples were fixed in 3% glutaraldehyde + 3% paraformaldehyde in 0.1 M cacodylate buffer (CB), pH 7.2. Samples were washed 3 times for 15 minutes in 0.1 M CB, then were processed for 1 hour on ice in a postfixation solution of 1.5% K_4_Fe(CN)_6_ + 2% OsO_4_ in 0.1 M CB. Samples were then washed 3 times in 0.1 M CB, and 3 times in 0.1 M Na_2_ + acetate buffer, pH 5.2, followed by en bloc staining for 1 hour in 2% uranyl acetate + 0.1 M Na_2_ + acetate buffer, pH 5.2. Samples were then processed overnight in an automated tissue processor, including dehydration from H_2_O through 30%, 50%, 70%, 80%, 90%, 95%, 100% EtOH, followed by 100% acetone. Samples were infiltrated with Spurr’s resin at a ratio of acetone/Spurr’s resin of 2:1 for 1 hour, 1:1 for 2 hours, and 1:2 for 16 hours, then absolute Spurr’s resin for 24 hours. After embedding and polymerization, samples were sectioned on an ultramicrotome (Leica).

#### Image quantification.

For FISH images, 3 technical replicates were stained, and, using the 20× objective with an artificial zoom on 5× on region of interest (crypt or villus), images were taken. mRNA molecules for each stain were then counted in each picture and recorded. Data were compiled and an unpaired 2-tailed *t* test was conducted between the groups.

For IF stains for TFF1 and CLDN18, 3 technical replicates were stained and a 20× objective image was taken. For each enteroid replicate, DAPI^+^ cells were counted, and cells positive for the stain of interest were counted and recorded. For TFF1, cells with cytoplasmic staining were counted as positive cells, and for CLDN18, cells with a complete membrane stain around DAPI^+^ nuclei were counted as positive cells. The percentage of enteroid sections expressing the stain of interest was calculated by counting the number of cells that are positive for the stain divided by the total number of DAPI^+^ cells multiplied by 100 per sample. A multiple comparisons 1-way ANOVA test was run to calculate statistical significance using GraphPad Prism software.

For relative intensity measurements on CDX2 stains, 5 enteroids per condition were outlined with the freehand tool in ImageJ (NIH). These enteroids were analyzed using the measure tool, and the IntDen for each enteroid was recorded and reported. A multiple comparisons 1-way ANOVA test was run to calculate statistical significance using GraphPad Prism software.

#### Enteroid shape quantifications.

To measure enteroid area, solidity, aspect ratio, circularity, and roundness, 6 enteroids per condition were grown 10 days for 1 passage. A 25× bright-field image of a single enteroid was outlined manually using the freehand selection tool in ImageJ, and shapes of the outlines were measured with measurements set to capture area and shape descriptors. For each condition, 6 enteroids were measured 3 times, and these measurements were graphed in Supplemental Figure 2A.

### Enteroid cultures and quantification

#### Isolating, establishing, and maintaining human fetal enteroids.

All enteroid lines described in this manuscript were generated from human tissue specimens obtained from the University of Washington Laboratory of Developmental Biology specifically for this study. Fresh human fetal epithelium was isolated and maintained exactly as previously described (46). The region of the duodenum that is posterior to the small intestinal entry point of the common bile duct (major duodenal papilla/papilla of Vater) was identified, and all experiments involving this tissue (i.e., for enteroid generation, to collect tissue for sequencing, or for histological processing for IF or FISH) were carried out with tissue from this area. We used this as a landmark so that the regions we collected were consistent across experiments and across different developmental stages. Briefly, duodenal tissue was cut into smaller pieces (~0.5–1 cm) and cut open longitudinally to expose the villi. To separate the epithelium, specimens then were incubated in dispase (StemCell Technologies, catalog 07923) for 30 minutes on ice in a Petri dish. Dispase then was removed and replaced with 100% fetal bovine serum (FBS) for 15 minutes on ice. To mechanically separate the tissue layers, 3 mL of Advanced DMEM/F12 (Gibco, catalog 12634-028) equal to the initial volume of FBS was added to the biopsy tissue before vigorously pipetting the mixture several times. Epithelial fragments then settled to the bottom of the Petri dish, where they were collected manually on a stereoscope by pipette. The epithelium was then washed with ice-cold Advanced DMEM/F12 and allowed to settle to the bottom of a 1.5 mL tube. The supernatant was withdrawn from the loose tissue pellet and replaced with Matrigel (Corning, catalog 354234). The Matrigel containing the isolated epithelium then was gently mixed before being pipetted into individual 50 μL droplets in a 24-well plate. The plate containing the droplets was then incubated at 37°C for at least 10 minutes to allow the Matrigel to solidify before adding media (see *Media composition* section below).

All experiments comparing media conditions were derived from the same fetal sample. At least 3 biological replicates of matched enteroids were grown in each media condition. Once enteroids were established, Matrigel droplets were dislodged from the culture plate and were pipetted in a Petri dish. Healthy enteroids were manually selected from the Petri dish under a stereoscope to be bulk-passaged through a 30G needle and embedded in Matrigel (Corning, catalog 354234). For single-cell passaging, healthy enteroids were manually selected under a stereoscope and dissociated with TrypLE Express (Gibco, catalog 12605-010) at 37°C for 10 minutes before filtering through 40 mm cell strainers. Cells were then counted using a hemocytometer, and 10,000 cells were embedded per Matrigel droplet. After 10 days, enteroids’ forming efficiency was calculated by taking the number of enteroids that had formed and dividing it by total number of cells seeded (10,000 cells).

#### Media composition.

Culture media consisted of LWRN conditioned media generated as previously described (46, 47) and combined with human basal media (Advanced DMEM/F12 [Gibco, catalog 12634-028]; Glutamax 4 mM [Gibco, catalog 35050-061]; HEPES 20 mM [Gibco, catalog 15630-080]; N2 Supplement [2×] [Gibco, catalog 17502-048]; B27 Supplement [2×] [Thermo Fisher Scientific, catalog 17504-044]; penicillin-streptomycin [2×] [Gibco, catalog 15140-122], *N*-acetylcysteine [2 mM] [MilliporeSigma, catalog A9165-25G], nicotinamide [20 mM] [MilliporeSigma, catalog N0636-061]). Complete media were composed of 25% LWRN and 75% human basal media to which recombinant human EGF (R&D Systems, catalog 236-EG, 100 ng/mL, 10 ng/mL, or 1 ng/mL) or recombinant human EREG (R&D Systems, catalog 5898-NR-050, 100 ng/mL, 10 ng/mL, or 1 ng/mL) was added.

#### Experimental design of enteroid cultures.

The enteroid experiments were designed and conducted to reduce batch effects in scRNA-Seq data and dual snRNA-Seq/ATAC-Seq data. All experiments comparing different treatment groups (i.e., EGF or EREG) were carried out in parallel, with experiments and treatments being conducted at the same time. Cells were harvested and dissociated into single-cell suspensions or single-nucleus suspensions in parallel (more details below). Since the 10x Chromium system allows parallel processing of multiple samples at a time, cells were captured (gel bead-in-emulsion) and processed (i.e., library prep) in parallel by the University of Michigan Advanced Genomics Core. All samples were sequenced across the same lane(s) on a NovaSeq 6000 for both transcriptional and epigenomic data.

#### Experimental design of ligand switch experiment.

The experiment outlined in Figure 4 was conducted as follows. Enteroids were generated as discussed above and allowed to grow for 2 passages (split approximately every 10 days) in 1 ng/mL EREG, 1 ng/mL EGF, or 100 ng/mL EGF. At the third passage, each enteroid sample was split and separated into new culture conditions of 1 ng/mL EREG, 1 ng/mL EGF, and 100 ng/mL EGF, making a total of 9 conditions. These enteroids were allowed to grow for 2 more passages. Then they were collected and flash-frozen for RT-qPCR analysis.

#### RNA extraction and RT-qPCR.

Three replicates from the same biological specimen were included in each analysis. mRNA was isolated using the MagMAX-96 Total RNA Isolation Kit (Thermo Fisher Scientific, catalog AM1830), and RNA quality and yield were measured on a NanoDrop 2000 spectrophotometer (Thermo Fisher Scientific) prior to cDNA synthesis. cDNA synthesis was performed using 100 ng RNA from each sample and using the SuperScript VILO cDNA Kit (Thermo Fisher Scientific, catalog 11754250). RT-qPCR was performed on a Step One Plus Real-Time PCR System (Thermo Fisher Scientific, catalog 43765592R) using QuantiTect SYBR Green PCR Kit (QIAGEN, catalog 204145). Expression of genes in the measurement of arbitrary units was calculated relative to RN18S using the following equation: 2^RN18S(CT)^^–^^GENE(CT)^ × 1,000.

### Single-cell experiments

#### Single-cell dissociation.

To dissociate enteroids to single cells, matched healthy enteroids to the same human sample in different media conditions were manually collected under a stereoscope. Following collection, dissociation enzymes and reagents from the Neural Tissue Dissociation Kit (Miltenyi Biotec, 130-092-628) were used, and all incubation steps were carried out in a refrigerated centrifuge prechilled to 10°C unless otherwise stated. All tubes and pipette tips used to handle cell suspensions were prewashed with 1% BSA in 1× HBSS to prevent adhesion of cells to the plastic. Tissue was treated for 15 minutes at 10°C with Mix 1 and then incubated for 10-minute increments at 10°C with Mix 2 interrupted by agitation by pipetting with a P1000 pipette until fully dissociated. Cells were filtered through a 70 μm filter coated with 1% BSA in 1× HBSS, spun down at 500*g* for 5 minutes at 10°C, and resuspended in 500 mL 1× HBSS (with Mg2^+^, Ca2^+^). Cells were spun down (500*g* for 5 minutes at 4°C) and washed twice by suspension in 2 mL of HBSS + 1% BSA, followed by centrifugation (500*g* for 5 minutes at 4°C). Cells were counted using a hemocytometer, then spun down and resuspended to reach a concentration of 1,000 cells/μL and kept on ice. Single-cell libraries were immediately prepared on the 10x Genomics Chromium by the University of Michigan Advanced Genomics Core facility with a target capture of 5,000 cells. A full, detailed protocol of tissue dissociation for scRNA-Seq can be found at http://www.jasonspencelab.com/protocols

#### Single-cell library preparation and transcriptome alignment.

All single-cell RNA-Seq sample libraries were prepared with the 10x Chromium Controller using v3 chemistry (10x Genomics, catalog 1000268). Sequencing was performed on a NovaSeq 6000 with targeted depth of 100,000 reads per cell. Default alignment parameters were used to align reads to the pre-prepared human reference genome (hg19) provided by the 10x Genomics Cell Ranger pipeline. Initial cell demultiplexing and gene quantification were also performed using the default 10x Genomics Cell Ranger pipeline.

#### Single-cell data analysis.

All scRNA-Seq analysis downstream of gene quantification was done using Scanpy (48) with the 10x Genomics Cell Ranger–derived gene-by-cell matrices. For primary human tissue sample analysis in Figure 1, 127-day and 132-day postconception duodenum (3 samples) and ileum (1 sample) were used. All samples were filtered to remove cells with fewer than 800 or greater than 3,500 genes or greater than 12,000 unique molecular identifier (UMI) counts per cell and less than 0.1 mitochondrial counts. De-noised data matrix read counts per gene were log-normalized prior to analysis. After log normalization, highly variable genes were identified and extracted, and batch correction was performed using the BBKNN algorithm. The normalized expression levels then underwent linear regression to remove effects of total reads per cell and cell cycle genes, followed by a *z* transformation. Dimension reduction was performed using principal component analysis (PCA) and then UMAP on the top 11 principal components (PCs) and 15 nearest neighbors for visualization on 2 dimensions. Clusters of cells within the data were calculated using the Louvain algorithm within Scanpy with a resolution of 0.3. Epithelial cells were identified using canonically expressed genes and extracted from a data matrix to include 1,009 intestinal epithelial cells from all samples. The extracted epithelial cell matrix then again underwent log normalization, variable gene extraction, *z* transformation, and dimension reduction to be displayed in the UMAP seen in Figure 1.

For Figure 2, the EREG-grown samples were filtered to remove cells with fewer than 1,200 or greater than 8,500 genes, or greater than 65,000 UMI counts per cell, and less than 0.1 mitochondrial counts. Data matrix read counts per gene were log-normalized prior to analysis. After log normalization, highly variable genes were identified and extracted. Data were then scaled by *z* transformation. Dimension reduction was performed using PCA and then UMAP on the top 10 PCs and 15 nearest neighbors for visualization. Clusters of cells within the data were calculated using the Louvain algorithm within Scanpy with a resolution of 0.5. The EGF-grown sample was filtered to remove cells with fewer than 1,250 or greater than 8,500 genes, or greater than 65,000 UMI counts per cell, and less than 0.1 mitochondrial counts. Data matrix read counts per gene were log-normalized prior to analysis. After log normalization, highly variable genes were identified and extracted. Data were then scaled by *z* transformation. Dimension reduction was performed using PCA and then UMAP on the top 10 PCs and 15 nearest neighbors for visualization. Clusters of cells within the data were calculated using the Louvain algorithm within Scanpy with a resolution of 0.4.

For Figure 3, all enteroid samples were filtered to remove cells with fewer than 1,200 or greater than 7,500 genes, or greater than 50,000 UMI counts per cell, and less than 0.1 mitochondrial counts. Data matrix read counts per gene were log-normalized prior to analysis. After log normalization, highly variable genes were identified and extracted. Data was then scaled by *z* transformation. Dimension reduction was performed using PCA and then UMAP on the top 12 PCs and 15 nearest neighbors for visualization. Clusters of cells within the data were calculated using the Louvain algorithm within Scanpy with a resolution of 0.3.

#### Cell scoring analysis.

Cells were scored based on expression of a set of 50 marker genes per epithelial tissue type. Gene lists were compiled based on the previously published top 50 most differentially expressed genes from in vivo human epithelial cells of the small intestine and stomach (33). See Supplemental Table 2 for gene lists. After obtaining the log-normalized and scaled expression values for the data set, scores for each cell were calculated as the average *z* score within each set of selected genes.

#### Receptor-ligand analysis.

In silico ligand-receptor analysis was performed using the CellChat R Package (https://github.com/sqjin/CellChat; commit ID c7cd04a) on the 127-day and 132-day human fetal intestine data sets. Data sets were imported and a Seurat object was created for each individual data set. These objects were then combined and filtered using the same parameters outlined in the *Single-cell data analysis* section above for Figure 1. This filtered objected was then normalized and scaled, and variable features were extracted. The data were then clustered, and cluster identities were annotated based on canonically expressed genes for each cell lineage (as shown in Supplemental Figure 1, E and F). These include neurons (*S100B*, *PLP1*, *STMN2*, *ELAVL4*), endothelial cells (*CDH5*, *KDR*, *ECSCR*, *CLDN5*), mesenchymal cells (*COL1A1*, *COL1A2*, *DCN*, *ACTG2*, *MYLK*), epithelial cells (*EPCAM*, *CDH1*, *CDX2*, *CLDN4*), immune cells (*PTPRC*, *HLA-DRA*, *ARHGDIB*, *CORO1A*), smooth muscle cells (*ACTA2*, *TAGLN*), subepithelial cells (*F3*, *PDGFRA*, *NRG1*), ISCs (*LGR5*, *OLFM4*), enterocytes (*FABP2*, *ALPI*, *RBP2*), BEST4^+^ enterocytes (*BEST4*, *SPIB*), goblet cells (*MUC2*, *SPDEF*, *DLL1*), tuft cells (*TRPM5*, *TAS1R3*), and enteroendocrine cells (*CHGA*, *NEUROD1*, *PAX6*, *ARX*, *REG4*). These identities were used as input for the CellChat analysis pipeline, and standard parameters and workflow outlined in CellChat documentation were used.

#### Endoderm atlas.

Reference map embedding to the Human Fetal Endoderm Atlas (33) was performed using the scoreHIO R Package (https://github.com/Camp-Lab/scoreHIO; commit ID 4d0820b). Enteroid samples were processed following the preprocessing steps outlined above in the *Single-cell data analysis* section and then put through the basic workflow outlined in the package to map enteroid cells onto the reference map and quantify their identity.

### Dual snRNA-Seq/ATAC-Seq experiments

#### Single-nucleus isolation and permeabilization.

Nuclei were isolated and permeabilized in accordance with 10x Genomics’ protocol for Nuclei Isolation from Complex Tissues for Single Cell Multiome ATAC + Gene Expression Sequencing. Briefly, tissue was minced into smaller fragments and then put into NP40 lysis buffer (Thermo Fisher Scientific, catalog PI28324). Tissue was homogenized with a pellet pestle 15 times by hand, then incubated in the lysis buffer for 5 minutes. The suspension was then passed through a 70 μm strainer followed by a 40 μm strainer (Thermo Fisher Scientific). The suspension was then centrifuged at 500*g* for 5 minutes at 4°C. The supernatant was removed, and the pellet was washed twice with PBS + 1% BSA and centrifuged at 500*g* for 5 minutes at 4°C after each wash. The pellet was then incubated in 0.1× lysis buffer and incubated for 2 minutes to permeabilize the sample. The sample was then centrifuged at 500*g* for 5 minutes at 4°C; supernatant was removed and resuspended in diluted nuclei buffer.

#### Single-nucleus/ATAC library preparation and transcriptome alignment.

All multiomics sample libraries were prepared with the Single Cell Multiome ATAC + Gene Expression v1 chemistry. Sequencing was performed on a NovaSeq 6000 with a targeted depth of 25,000 reads per nucleus for both ATAC and RNA. Default alignment parameters were used to align reads to the pre-prepared hg38 human reference genome provided by the 10x Genomics Cell Ranger ARC pipeline. Initial cell demultiplexing and gene quantification were also performed with the default 10x Genomics Cell Ranger ARC pipeline.

#### Single-nucleus analysis.

All multiomic analysis downstream of gene quantification were done using Seurat and the Signac extension with the 10x Cell Ranger ARC-derived gene-by-cell matrices. For sample analysis in Figures 4 and Supplemental Figure 2A, all samples were filtered to remove cells with greater than 100 but fewer than 15,000 UMI counts per cell for the RNA assay and greater than 100 or fewer than 45,000 UMIs for the ATAC assay. A transcriptional start site enrichment score of 0.5 or over was also used in filtering. Next, peak calling was performed using MACS2 (49). Normalization of gene expression and dimension reduction was performed using PCA. DNA accessibility assay was processed by latent semantic indexing, and a WKNN graph was calculated representing a weighted combination of RNA- and ATAC-Seq modalities. We used this graph to perform cell type clustering and visualized it using a UMAP visualization. Epithelial cells were identified based on their expression of the pan-epithelial marker *CDH1* and lack of expression of pan-mesenchymal marker *VIM*. From here, motif analysis and peak visualization were performed.

### Data and code availability statement

Sequencing data generated and used by this study are deposited at EMBL-EBI ArrayExpress. Data sets for human fetal intestine (ArrayExpress: E-MTAB-9489, https://www.ebi.ac.uk/arrayexpress/experiments/E-MTAB-9489/, and previously published work: ref. 13), scRNA-Seq of human fetal intestinal enteroids (ArrayExpress: E-MTAB-11912, https://www.ebi.ac.uk/arrayexpress/experiments/E-MTAB-11912/), and dual snRNA/snATAC-Seq multiomic data of human fetal intestinal enteroids (ArrayExpress: E-MTAB-11908, https://www.ebi.ac.uk/arrayexpress/experiments/E-MTAB-11908/) have been deposited. Code used to process raw data can be found at https://github.com/jason-spence-lab/Childs_2022 (commit ID c8bd5a7).

### Statistics

For statistical analysis, data were collected and compiled using GraphPad Prism software. For significance tests between 2 groups, an unpaired 2-tailed Welch’s *t* test was conducted between the groups using a *P* < 0.05. For significance testing between 3 or more groups, an ordinary 1-way ANOVA with multiple comparisons test was run using a *P* < 0.05 significance cutoff.

### Study approval

Normal human intestinal tissue was obtained from the University of Washington Laboratory of Developmental Biology. All human tissue used in this work was deidentified, and experiments were conducted with approval from the University of Michigan Institutional Review Board and the University of Washington Institutional Review Board.

## Author contributions

CJC and JRS conceived the study. JRS supervised the research. CJC and OPC performed computational analyses. CJC, JHW, OPC, QY, JGC, MPV, and JRS interpreted computational results and provided support. AW, EMH, YHT, and MMC developed tissue dissociation methods and generated scRNA-Seq data. CJC developed tissue dissociation method for multiomic analysis and the joint sequencing data. EMH, CWS, CJC, CJZ, AV, and JZS performed and analyzed all staining experiments and imaging. CWS, YHT, AW, and CJC performed enteroid experiments. CJC, CWS, YHT, AW, MMC, MKE, and EMH analyzed and interpreted enteroid experiments. RKZ, BP, CT, ODK, and IG provided critical material resources for this work. CJC and EMH assembled figures. CJC, EMH, and JRS wrote the manuscript. CJC, EMH, CWS, YHT, AW, JHW, MKE, and CL contributed methods. All authors edited, read, and approved the manuscript.

## Supplementary Material

Supplemental data

Supplemental table 1

Supplemental table 2

Supplemental table 3

## Version 1 02/23/2023

In-Press Preview

## Version 2 03/22/2023

Electronic publication
