# Supplementary material for: EPIREGULIN creates a developmental niche for spatially organized human intestinal enteroids
Source: JCI Insight. 2023 Mar 22;8(6):e165566. doi: 10.1172/jci.insight.165566 (PMC10070114; doi:10.1172/jci.insight.165566)
Supplement: Supplemental data [file jciinsight-8-165566-s005.pdf]

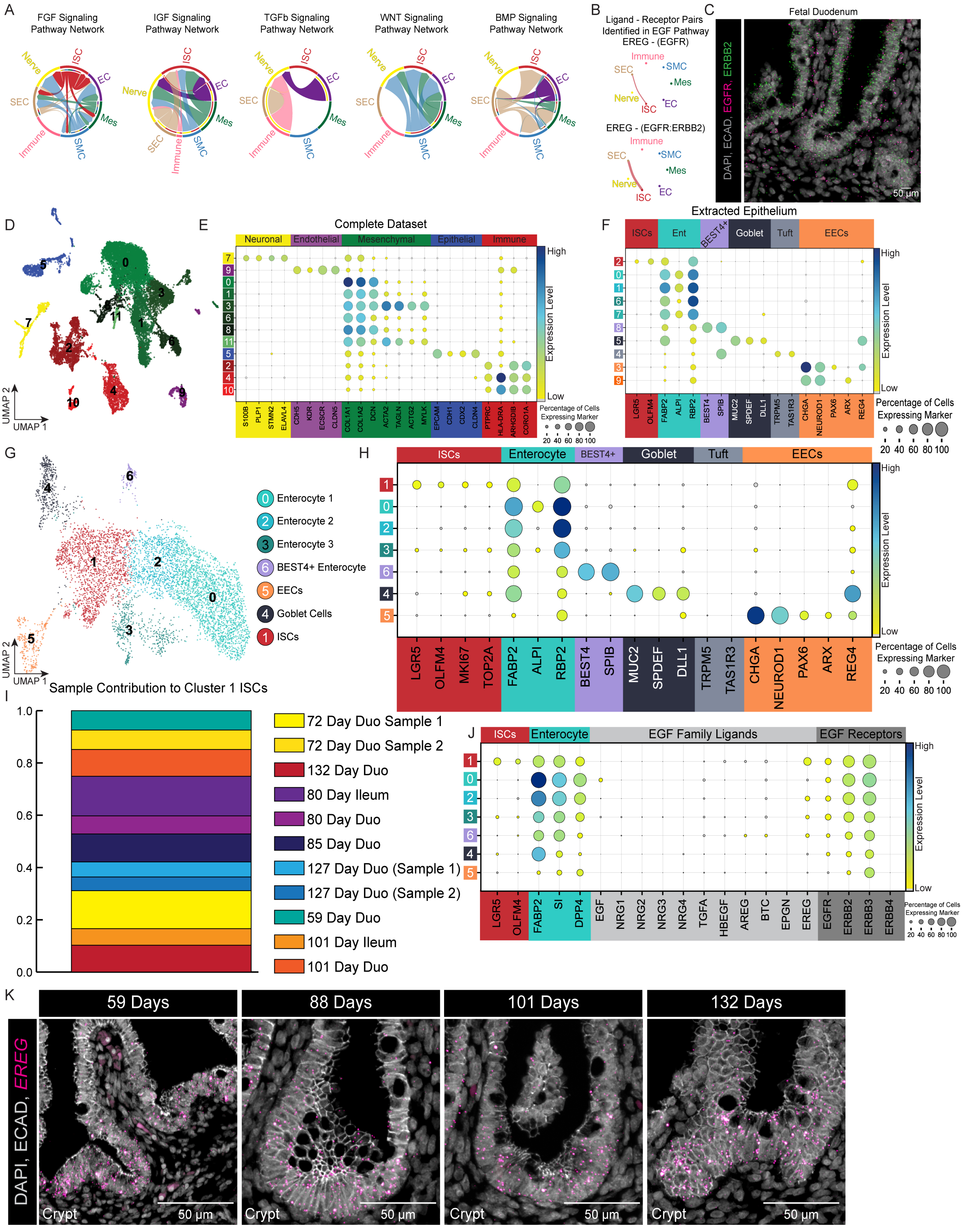
A
FGF Signaling Pathway Network
IGF Signaling Pathway Network
TGFb Signaling Pathway Network
WNT Signaling Pathway Network
BMP Signaling Pathway Network
Nerve
ISC
EC
Mes
SMC
Immune
SEC
B
Ligand - Receptor Pairs Identified in EGF Pathway
EREG - (EGFR)
EREG - (EGFR:ERBB2)
C
Fetal Duodenum
DAPI, ECAD, EGFR, ERBB2
50 µm
D
UMAP 1
UMAP 2
E
Complete Dataset
Neuronal
Endothelial
Mesenchymal
Epithelial
Immune
S100B
PLP1
STMN2
ELAVL4
CDH5
KDR
ESCSR
CLDN5
COL1A1
COL1A2
DCN
ACTA2
TAGLN
ACTG2
MYLK
EPCAM
CDH1
CDX2
CLDN4
PTPRC
HLA-DRA
ARHGDIB
CORO1A
High
Expression Level
Low
Percentage of Cells Expressing Marker
20 40 60 80 100
F
Extracted Epithelium
ISCs
Ent
BEST4+
Goblet
Tuft
EECs
LGR5
OLFM4
FABP2
ALPI
RBP2
BEST4
SPIB
MUC2
SPDEF
DLL1
TRPM5
TAS1R3
CHGA
NEUROD1
PAX6
ARX
REG4
G
Enterocyte 1
Enterocyte 2
Enterocyte 3
BEST4+ Enterocyte
EECs
Goblet Cells
ISCs
H
ISCs
Enterocyte
BEST4+
Goblet
Tuft
EECs
LGR5
OLFM4
MKI67
TOP2A
FABP2
ALPI
RBP2
BEST4
SPIB
MUC2
SPDEF
DLL1
TRPM5
TAS1R3
CHGA
NEUROD1
PAX6
ARX
REG4
I
Sample Contribution to Cluster 1 ISCs
1.0
0.8
0.6
0.4
0.2
0.0
72 Day Duo Sample 1
72 Day Duo Sample 2
132 Day Duo
80 Day Ileum
80 Day Duo
85 Day Duo
127 Day Duo (Sample 1)
127 Day Duo (Sample 2)
59 Day Duo
101 Day Ileum
101 Day Duo
J
ISCs
Enterocyte
EGF Family Ligands
EGF Receptors
LGR5
OLFM4
FABP2
SI
DPP4
EGF
NRG1
NRG2
NRG3
NRG4
TGFA
HBEGF
AREG
BTC
EPGN
EREG
EGFR
ERBB2
ERBB3
ERBB4
K
59 Days
88 Days
101 Days
132 Days
DAPI, ECAD, EREG
Crypt
50 µm

Supplemental Figure 1. Whole Tissue Cell Cluster Annotation and Receptor-Ligand Analysis of Developing Human Small Intestine at Single Cell Resolution. Related to Figure 1.

(A) Chord diagram of predicted FGF, IGF, TGFb, WNT, and BMP signaling events between intestinal stem cells and additional non-epithelial lineages. Cell type abbreviations are as follows: SEC (subepithelial cells), SMC (smooth muscle cells), Mes (mesenchyme), ISC (intestinal stem cells), EC (endothelial cells).

(B) Diagram showing the contribution of ligand-receptor pairing to chord diagram in Figure 1D. EREG was the only ligand predicted in the stem cell cluster.

(C) Co-FISH/IF staining for *EGFR* (pink), *ErbB2* (green), and ECAD (grey) in human fetal duodenum (127-day).

(D) UMAP visualization of entire merged fetal datasets colored broadly by cell class (neurons; yellow, endothelial; purple, mesenchyme; green, epithelium; blue, and immune; red) datasets include 2 biological replicates, ages 127-day (two duodenal samples) and 132-day (one duodenal and one ileum) with 18,100 cells total.

(E) Dot plot of entire fetal datasets highlighting expression of canonical lineage genes that were used for cluster annotation.

(F) Dot plot illustrating expression of canonical epithelial lineage markers within fetal epithelial subclusters.

(G) UMAP visualization of extracted epithelial clusters from entire fetal intestine dataset(29). Genes used for extraction matched those used in (E).

(H) Dot plot illustrating expression of canonical epithelial lineage markers within fetal epithelial subclusters.

(I) Bar graph of sample contribution to ISC cluster (cluster 1) of the 11 samples included in this analysis.

(J) Dot plot visualization of stem cell markers *(LGR5, OLFM4*), enterocyte markers (*FABP2, SI, DPP4*), EGF ligands, and EGF family receptors among human fetal epithelial datasets.

(K) Co-FISH/IF staining for *EREG* (pink), DAPI (grey), and ECAD (grey) in the crypts of the human fetal duodenum at select timepoint across developmental time.

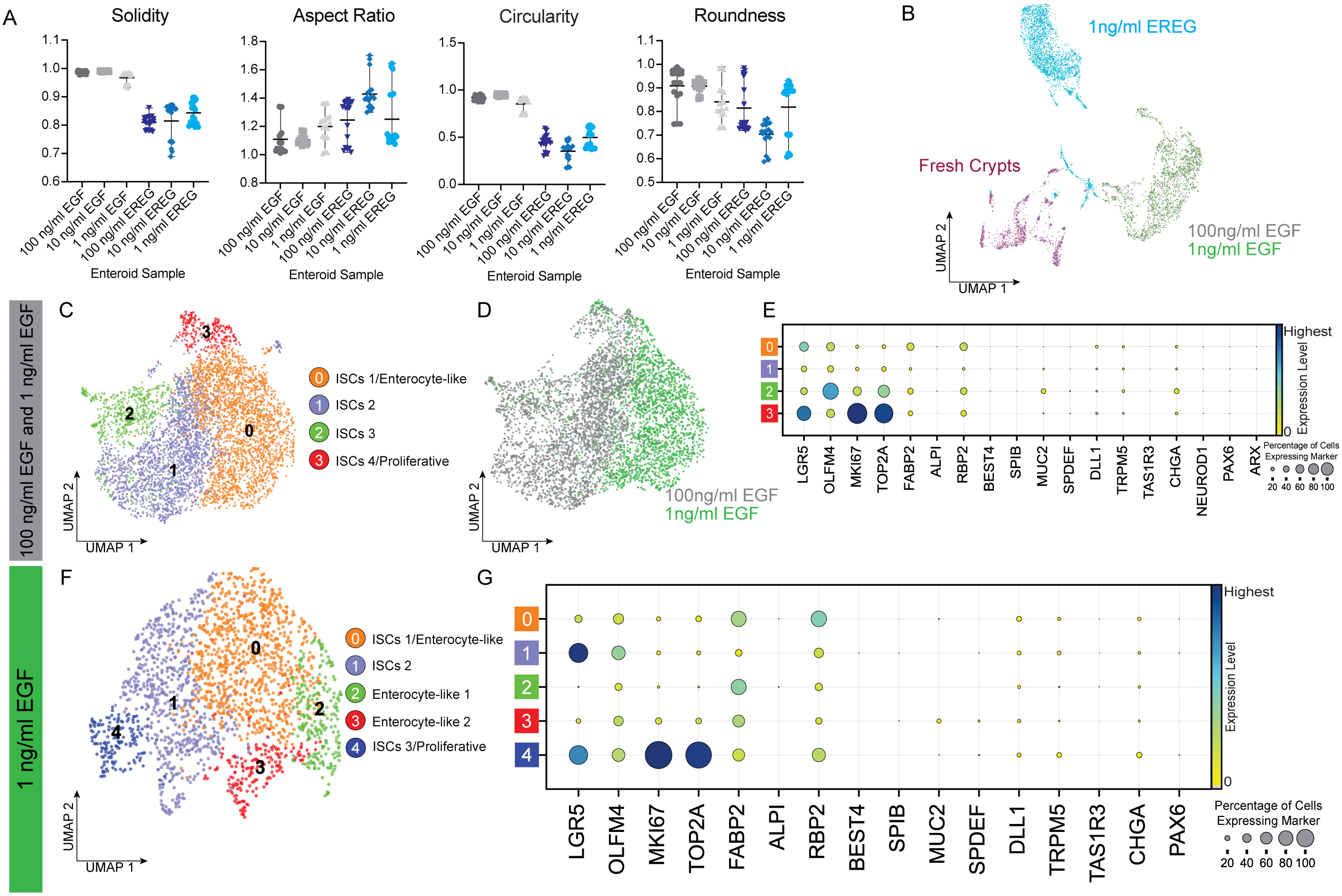

A
Solidity
Aspect Ratio
Circularity
Roundness
100 ng/ml EGF
10 ng/ml EGF
1 ng/ml EGF
100 ng/ml EREG
10 ng/ml EREG
1 ng/ml EREG
Enteroid Sample
B
1ng/ml EREG
Fresh Crypts
100ng/ml EGF
1ng/ml EGF
UMAP 1
UMAP 2
100 ng/ml EGF and 1 ng/ml EGF
C
ISCs 1/Enterocyte-like
ISCs 2
ISCs 3
ISCs 4/Proliferative
D
100ng/ml EGF
1ng/ml EGF
E
Highest
Expression Level
LGR5
OLFM4
MKI67
TOP2A
FABP2
ALPI
RBP2
BEST4
SPIB
MUC2
SPDEF
DLL1
TRPM5
TAS1R3
CHGA
NEUROD1
PAX6
ARX
Percentage of Cells Expressing Marker
20 40 60 80 100
1 ng/ml EGF
F
ISCs 1/Enterocyte-like
ISCs 2
Enterocyte-like 1
Enterocyte-like 2
ISCs 3/Proliferative
G
Highest
Expression Level
Percentage of Cells Expressing Marker
20 40 60 80 100

Supplemental Figure 2. Quantification and Additional scRNA-seq Analysis of EGF-grown Enteroids. Related to Figure 2.

(A) Quantification of solidity, aspect ratio, circularity, and roundness of enteroids grown in varying doses of EGF or EREG. Six enteroids grown for one passage to 10 days were measured three times. See methods section for further explanation on calculations.

(B) Weighed nearest neighbors (representing joint RNA and ATAC data) UMAP visualization colored by sample (fresh crypts; red, 1 ng/ml EREG; blue, 1 ng/ml EGF; green, and 100 ng/ml EGF; grey). Overlap of EGF conditions suggests transitional and epigenomic similarity.

(C) UMAP visualization of scRNA-seq data (no epigenomic data included) of 1 ng/ml EGF-grown enteroids and 100 ng/ml EGF-grown enteroids with 5,326 cells total in analysis.

(D) UMAP visualization of sample distribution between 1 ng/ml EGF (green) and 100 ng/ml EGF (grey) enteroids.

(E) Dot plot of canonically illustrating expression of canonical epithelial lineage markers within combined enteroids analysis.

(F) UMAP visualization of scRNA-seq data (no epigenomic data included) of 1 ng/ml EGF-grown enteroids alone with 2,099 cells total in analysis.

(G) Dot plot of canonically illustrating expression of canonical epithelial lineage markers within 1 ng/ml EGF-grown enteroids alone.

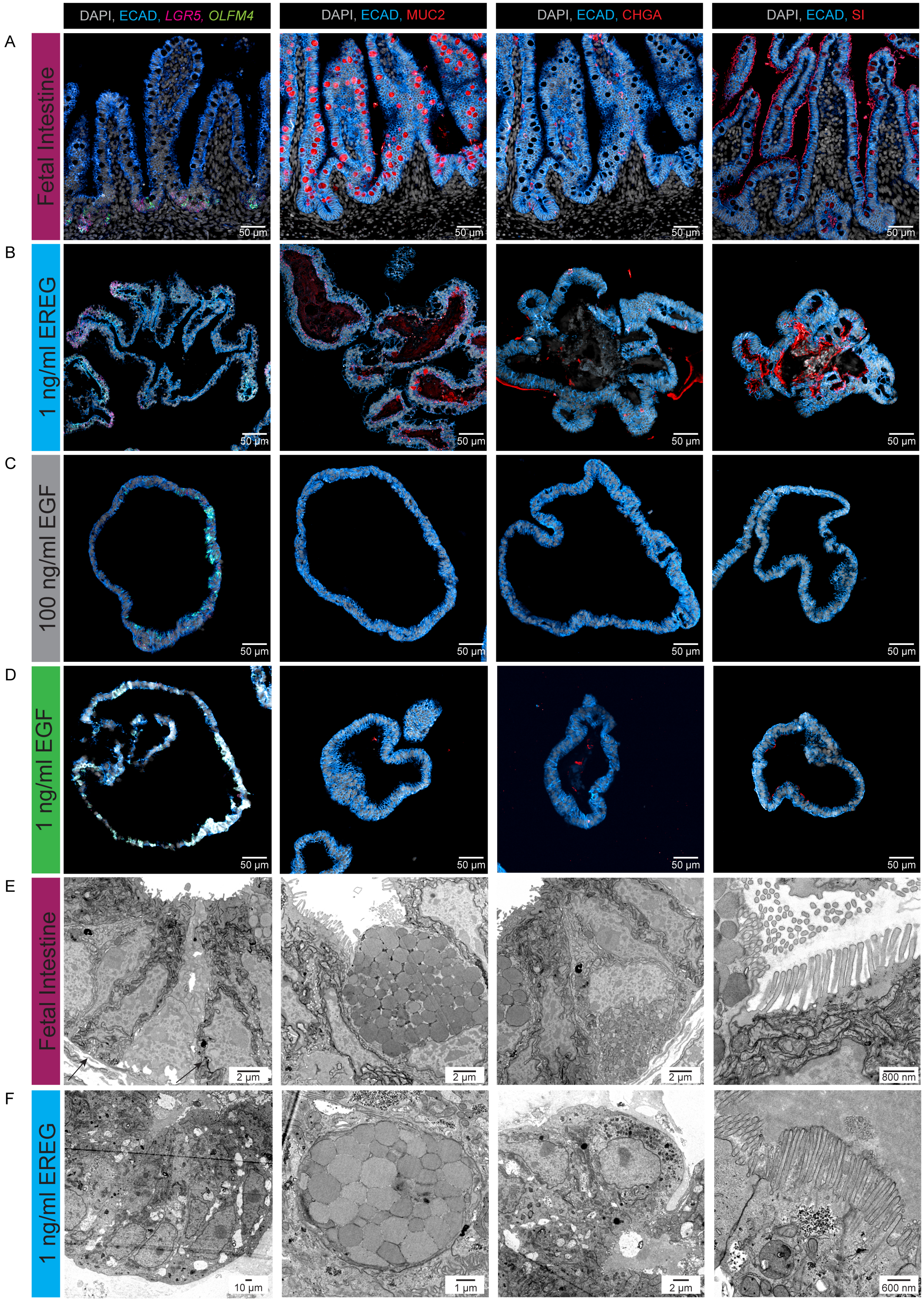

DAPI, ECAD, LGR5, OLFM4
DAPI, ECAD, MUC2
DAPI, ECAD, CHGA
DAPI, ECAD, SI
A
Fetal Intestine
B
1 ng/ml EREG
C
100 ng/ml EGF
D
1 ng/ml EGF
E
Fetal Intestine
F
1 ng/ml EREG
50 µm
2 µm
800 nm
10 µm
1 µm
600 nm

Supplemental Figure 3. Additional Staining of EREG and EGF-grown Enteroids. Related to Figure 2.

(A) FISH and immunofluorescence staining in fetal intestine (127-day) tissue sections for stem cells *(LGR5, OLFM4*), goblet cells (MUC2), enteroendocrine cells (CHGA), and brush border of enterocytes (SI). Middle panels show the same image with MUC2 and CHGA channels split for visual clarity.

(B) FISH and immunofluorescence staining of 1 ng/ml EREG-grown enteroids specimens for stem cells *(LGR5, OLFM4*), goblet cells (MUC2), enteroendocrine cells (CHGA), and brush border of enterocytes (SI).

(C) FISH and immunofluorescence staining of 100 ng/ml EGF-grown enteroids specimens for stem cells *(LGR5, OLFM4*), goblet cells (MUC2), enteroendocrine cells (CHGA), and brush border of enterocytes (SI).

(D) FISH and immunofluorescence staining of 1 ng/ml EGF-grown enteroids specimens for stem cells *(LGR5, OLFM4*), goblet cells (MUC2), enteroendocrine cells (CHGA), and brush border of enterocytes (SI).

(E-F) TEM imaging of human fetal intestine (89-day) (E) specimens and 1 ng/ml EREG-grown enteroids (F). Intracellular characteristics were used to classify the presence of stem cells (black arrows), goblet cells, enteroendocrine cells, and brush border of enterocytes. Note: First and third panel in (E) are separate images taken from the same region of tissue.

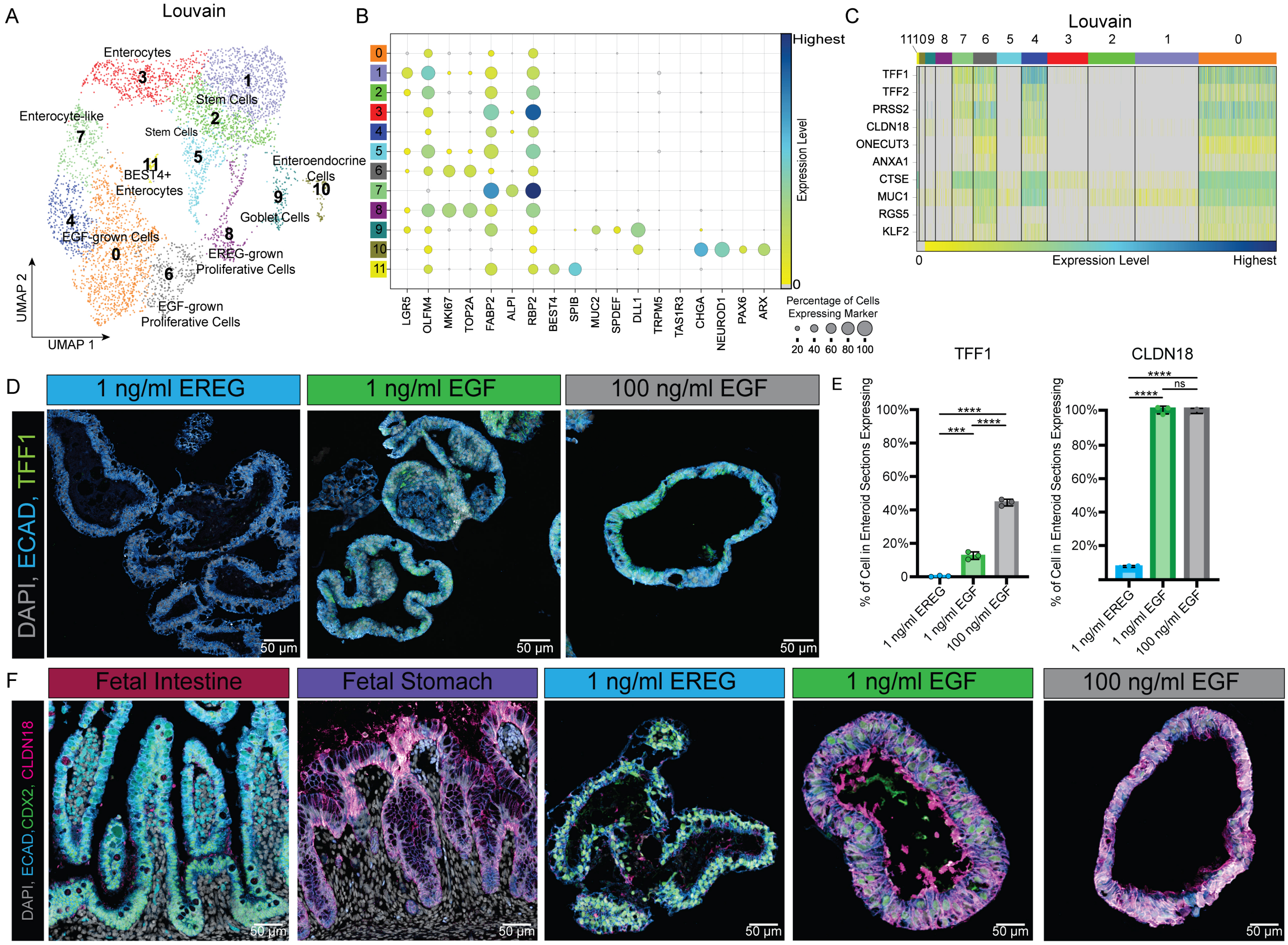
A
Louvain
Enterocytes
Stem Cells
Enterocyte-like
Stem Cells
BEST4+ Enterocytes
Enteroendocrine Cells
Goblet Cells
EGF-grown Cells
EREG-grown Proliferative Cells
EGF-grown Proliferative Cells
UMAP 1
UMAP 2
B
Highest
Expression Level
LGR5
OLFM4
MKI67
TOP2A
FABP2
ALPI
RBP2
BEST4
SPIB
MUC2
SPDEF
DLL1
TRPM5
TAS1R3
CHGA
NEUROD1
PAX6
ARX
Percentage of Cells Expressing Marker
20 40 60 80 100
C
Louvain
TFF1
TFF2
PRSS2
CLDN18
ONECUT3
ANXA1
CTSE
MUC1
RGS5
KLF2
Expression Level
Highest
D
1 ng/ml EREG
1 ng/ml EGF
100 ng/ml EGF
DAPI, ECAD, TFF1
50 μm
E
TFF1
CLDN18
% of Cell in Enteroid Sections Expressing
1 ng/ml EREG
1 ng/ml EGF
100 ng/ml EGF
ns
F
Fetal Intestine
Fetal Stomach
1 ng/ml EREG
1 ng/ml EGF
100 ng/ml EGF
DAPI, ECAD,CDX2, CLDN18
50 μm

Supplemental Figure 4. Cell Cluster Annotation of Enteroid Samples and Ectopic Stomach Expression by scRNA-seq and Immunofluorescence Imaging. Related to Figure 3.

(A) UMAP visualization of Louvain clustering of 1 ng/ml EREG and 100 ng/ml EGF enteroids. Clusters annotated using canonically expressed marker genes when possible, otherwise growth condition is noted in cluster name (i.e. EGF-grown cells).

(B) Dot plot visualization of canonically expressed marker gene used to annotated Louvain clustering in (A). These include stem cells (*LGR5, OLFM4*), proliferative cells (*MKI67, TOP2A*), enterocytes (*FABP2, ALPI, RBP2*), BEST4+ enterocytes (*BEST4, SPIB*), goblet cells (*MUC2, SPDEF, DLL1*), tuft cells (*TRPM5, TAS1R3*), and enteroendocrine (*CHGA, NEUROD1, PAX6, ARX*).

(C) Heatmap showing ectopic stomach gene expression in Louvain clusters with most expression occurring in EGF-grown cell clusters (0, 4, 6, 7).

(D) 2D immunofluorescence staining for stomach marker TFF1 (green) counterstained with ECAD (blue) and DAPI (grey) in 1 ng/ml EREG-grown enteroid (passage 1 day 10), 1 ng/ml EGF-grown enteroid (passage 1 day 10), and 100 ng/ml EGF-grown enteroid (passage 1 day 10). Controls in human fetal intestine (127-day), human fetal stomach (132-day) can be found in Figure 3.

(E) Quantification of TFF1 and CLDN18 immunofluorescence images. Statistical significance was determined with a one-way ANOVA with multiple comparisons using the GraphPad Prism software (TFF1: 1 ng/ml EREG to 1 ng/ml EGF adjusted p-value= 0.0003; 1 ng/ml EREG to 100 ng/ml EGF adjusted p-value=<0.0001); 1 ng/ml EGF to 100 ng/ml EGF adjusted p-value=<0.0001. CLDN18: 1 ng/ml EREG to 1 ng/ml EGF adjusted p-value=<0.0001; 1 ng/ml EREG to 100 ng/ml EGF adjusted p-value=<0.0001); 1 ng/ml EGF to 100 ng/ml EGF adjusted p-value=<0.9992).

(F) 2D immunofluorescence staining for stomach marker CLDN18 (pink) and intestinal marker CDX2 (green) counterstained with ECAD (blue) and DAPI (grey) in human fetal intestine (127-day), human fetal stomach (132-day), 1 ng/ml EREG-grown enteroid (passage 1 day 10), 1 ng/ml EGF-grown enteroid (passage 1 day 10), and 100 ng/ml EGF-grown enteroid (passage 1 day 10).

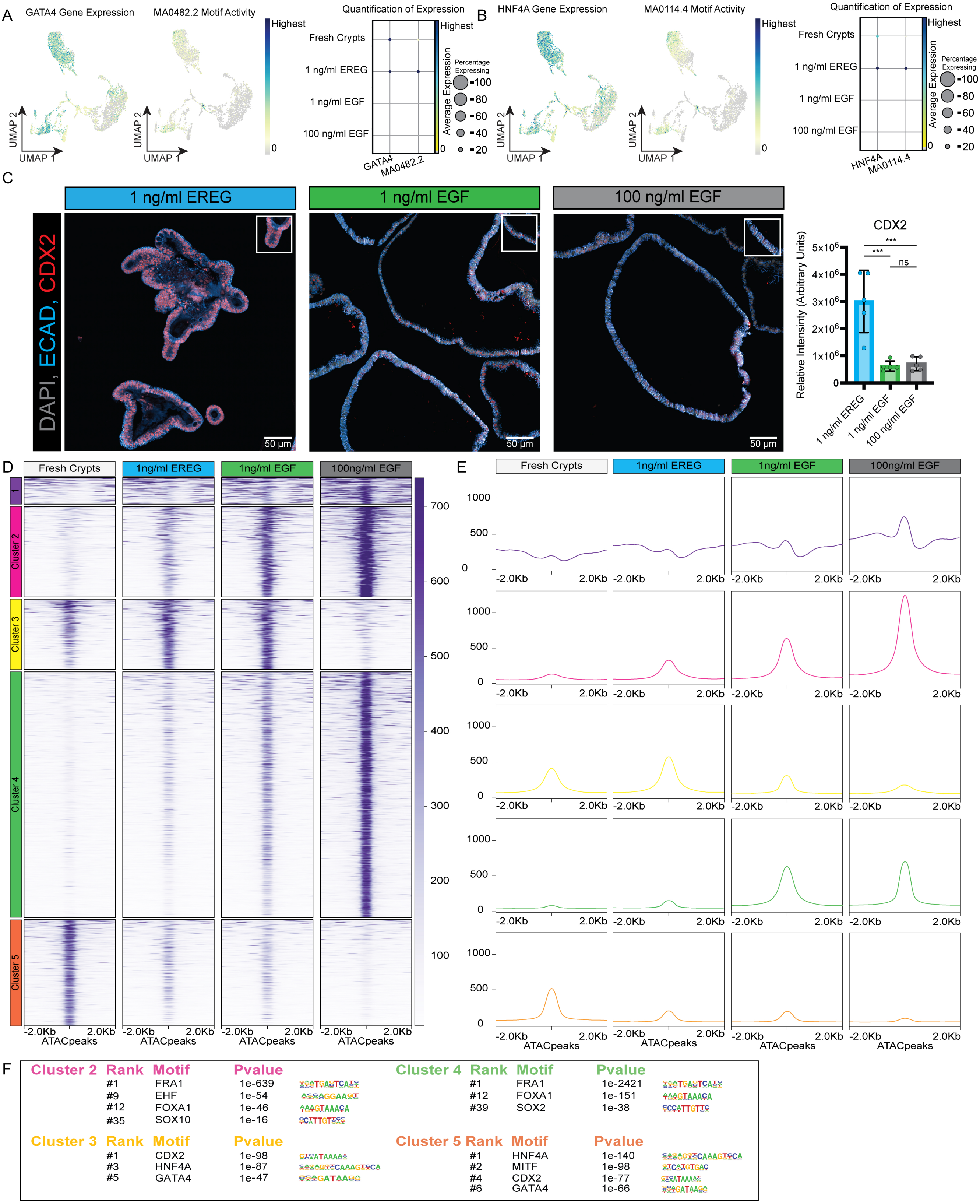
A
GATA4 Gene Expression
MA0482.2 Motif Activity
Highest
0
UMAP 1
UMAP 2
Quantification of Expression
Fresh Crypts
1 ng/ml EREG
1 ng/ml EGF
100 ng/ml EGF
GATA4
MA0482.2
Average Expression
Percentage Expressing
100
80
60
40
20
B
HNF4A Gene Expression
MA0114.4 Motif Activity
HNF4A
MA0114.4
C
DAPI, ECAD, CDX2
1 ng/ml EREG
1 ng/ml EGF
100 ng/ml EGF
50 μm
CDX2
Relative Intensity (Arbitrary Units)
5x10^6
4x10^6
3x10^6
2x10^6
1x10^6
0
***
***
ns
D
Fresh Crypts
1ng/ml EREG
1ng/ml EGF
100ng/ml EGF
1
Cluster 2
Cluster 3
Cluster 4
Cluster 5
-2.0Kb
2.0Kb
ATACpeaks
700
600
500
400
300
200
100
E
1000
500
0
F
Cluster 2 Rank Motif Pvalue
#1 FRA1 1e-639
#9 EHF 1e-54
#12 FOXA1 1e-46
#35 SOX10 1e-16
Cluster 3 Rank Motif Pvalue
#1 CDX2 1e-98
#3 HNF4A 1e-87
#5 GATA4 1e-47
Cluster 4 Rank Motif Pvalue
#1 FRA1 1e-2421
#12 FOXA1 1e-151
#39 SOX2 1e-38
Cluster 5 Rank Motif Pvalue
#1 HNF4A 1e-140
#2 MITF 1e-98
#4 CDX2 1e-77
#6 GATA4 1e-66

Supplemental Figure 5. Characterization of Multiomic Analysis and k-means Clustering. Related to Figure 4.

(A) UMAP visualization of GATA4 gene expression and motif activity of MA0482.2 with dot plot quantification.
(B) UMAP visualization of HNF4A gene expression and motif activity of MA0114.4with dot plot quantification.
(C) 2D immunofluorescence staining for intestinal marker CDX2 (red) counterstained with ECAD (blue) and DAPI (grey) in 1 ng/ml EREG-grown enteroid (passage 1 day 10), 1 ng/ml EGF-grown enteroid (passage 1 day 10), and 100 ng/ml EGF-grown enteroid (passage 1 day 10). Quantification of signal intensity is reported with 5 technical replicates of individual enteroids measured.
(D) K-means clustering of peaks in control fresh crypts, 1 ng/ml EREG, 1 ng/ml EGF, and 100 ng/ml EGF. Graph represents a heatmap of accessibility in these regions.
(E) Graphical schematic summary of overall pattern of peaks shown in (E).
(F) Motif analysis of clusters of interest from K-means clustering in (E) including motif and P-value. Full list of ranked motifs can be found in Table S3.
